# Overview of Research on Essential Oils of *Zanthoxylum bungeanum*: Composition, Activity, Applications, and Challenges

**DOI:** 10.3390/ph19030473

**Published:** 2026-03-13

**Authors:** Qing Du, Yuwan Diao, Yu Meng, Zihan Wang, Jing Zhang, Tingting Wu, Qiaoyi Huang, Xiaoying Huang, Ming Yang

**Affiliations:** State Key Laboratory for the Modernization of Classical and Famous Prescriptions of Chinese Medicine, Innovation and Entrepreneurship College, Jiangxi University of Chinese Medicine, Nanchang 330006, China; 20192019@jxutcm.edu.cn (Q.D.); jing.zhang@jxutcm.edu.cn (J.Z.); jxutcm0689@163.com (X.H.)

**Keywords:** *Zanthoxylum bungeanum*, volatile oil, chemical composition, influencing factors, pharmacological activity, action mechanisms, application

## Abstract

As the main active component of *Zanthoxylum bungeanum*, its volatile oil (ZEO) exhibits diverse pharmacological activities, including insecticidal, antibacterial, anti-inflammatory, and anti-tumor effects. These properties support its traditional functions, such as “expelling worms” and “warming the Middle Jiao to alleviate pain and relieve itching.” However, modern research mainly validates individual components or effects, leaving notable gaps in understanding this complex system. This review integrates research on ZEO, summarizing its composition, influencing factors, and mechanisms of action. By framing the “composition–activity–mechanism–application” continuum, this review analyzes the basis for the holistic, multi-component, multi-target therapeutic model of traditional Chinese medicine (TCM). It clarifies the core TCM principles of pharmacological symbiosis and synergy through formula compatibility. These insights form a theoretical basis for further development and wider application of ZEO in fields such as medicine, food, and daily chemical products.

## 1. Introduction

Zanthoxylum is a deciduous shrub or small tree belonging to the genus *Zanthoxylum* within the *Rutaceae* family. In China, *Zanthoxylum* species have a long history of application as a traditional spice, especially known for their unique numbing flavor and aromatic properties. China possesses abundant *Zanthoxylum* germplasm resources, and its medicinal base is mainly the dried mature pericarp of *Zanthoxylum bungeanum* Maxim. or *Z. schinifolium* Sieb. et Zucc. of the Rutaceae family [1]. Historically, “Qin jiao” (Shanxi and other places) and “Shu jiao” (Sichuan and other places) have long been famous. The use of *Zanthoxylum* in food and medicine dates back more than 2000 years. Shennong’s Classic of Materia Medica records that “*Zanthoxylum* tastes pungent and is warm in nature. It mainly treats wind-evil qi and can cure cough, reverse qi, cold-damp arthralgia and other symptoms” [2]. In the Eastern Han Dynasty, Zhang Zhongjing’s Synopsis of Prescriptions of the Golden Chamber includes the Dajianzhong Decoction, which uses *Zanthoxylum bungeanum* as the principal drug to warm the middle energizer and dispel cold, primarily treating cold pain in the epigastrium and abdomen [1]. Beyond historical materia medica, the modern Pharmacopoeia of the People’s Republic of China (2025 Edition) states that *Zanthoxylum* “warms the middle-energizer to relieve pain, kills parasites and relieves itching” and is indicated for epigastrium and abdomen, vomiting and diarrhea, and abdominal pain associated with parasitic infestation.

ZEO, the primary material basis of *Zanthoxylum*, concentrates the plant’s characteristic aroma and exhibits diverse pharmacological activities. Modern studies indicate that ZEO is a complex mixture dominated by monoterpenes and sesquiterpenoids (for example, limonene and β-myrcene) and by alcohols (for example, linalool), with trace amounts of esters, ketones, aldehydes, and other constituents that together form a multifaceted chemical system [3]. These constituents confer multiple bioactivities on ZEO, including anti-inflammatory, antibacterial, and antioxidant effects, which likely represent the core material basis for *Zanthoxylum*’s traditional uses of “warming the middle-energizer to relieve pain, killing parasites and relieving itching.” With regard to the effect described as “warming the middle and relieving pain,” the oil’s anti-inflammatory and analgesic properties can mitigate inflammatory responses and associated pain arising from factors such as invasion of cold pathogens or qi stagnation and blood stasis [4]. Inflammation typically produces local redness, swelling, heat, pain, and other pathological features; ZEO suppresses the inflammatory cascade and, through actions on nociceptive pathways, reduces nociceptor sensitivity, thereby relieving epigastric and abdominal cold pain [5]. Antimicrobial activity underlies its “insecticidal and antipruritic” effects. Because skin and gastrointestinal parasites and microbial infections frequently cause itching and discomfort, ZEO inhibition of various pathogens limits their proliferation and thereby reduces infection-associated pruritus and intestinal parasitic abdominal pain. Concurrently, its antioxidant activity moderates oxidative stress, preserves normal cellular function, and promotes tissue repair, all of which together support the combined actions of “warming the middle and relieving pain, and killing worms to relieve itching.”

Although the pharmacological activities of ZEO—including anti-inflammation, anti-bacterial, and antioxidant activities, are closely related to the efficacy of “warming the middle-energizer to relieving pain, killing parasites and relieving itching”, current investigations remain insufficient and limited. Most research focuses on the extraction of volatile oils and the analysis of numbing-taste components, whereas insufficient attention has been paid to the material basis underlying its traditional efficacies and the multi-component synergistic mechanism. Therefore, it is of great significance to write a research progress report on ZEO. It can not only systematically review the current research status and identify gaps, but also provide ideas for revealing its medicinal value and developing related products, thereby promoting deeper application of ZEO in medicine, food, and related fields.

**Figure 1:**
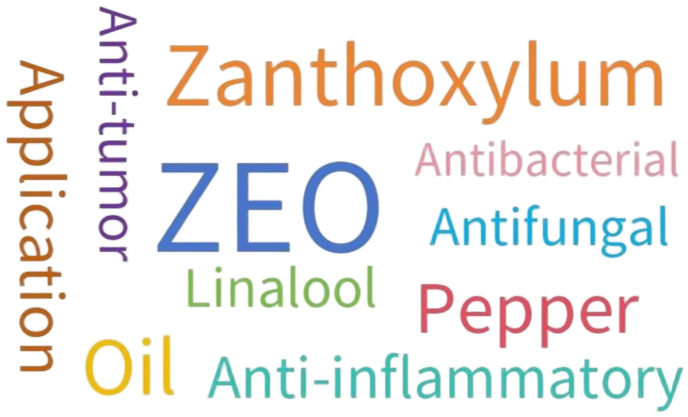
Word cloud of core research keywords for ZEO. This visualization intuitively presents its botanical source (*Zanthoxylum*), major active component (linalool), key biological activities (antitumor, antibacterial, antifungal, anti-inflammatory), and research hotspots such as application prospects.

This review comprehensively synthesizes the chemical composition of ZEO, factors influencing its composition, pharmacological properties and related mechanisms of action, as well as its future application prospects. The objective is to construct a systematic knowledge framework, clarify the scientific value of ZEO, and provide potential guidance for its industrial development and clinical use. The word cloud depicting the main pharmacological effects of ZEO is shown in Figure 1.

Literature retrieval and synthesis proceeded as follows: (1) Preliminary searches used the main keywords, specifically, “medicinal plants”, “essential oils”, “*Zanthoxylum bungeanum*”, “essential oil components”, “pharmacological effects”, and “applications” in databases such as PubMed, Web of Science, Science Direct, Scopus, Embase, SpringerLink, Scifinder, CNKI, Wanfang Data, and other databases for the period almost from 2015 to the present; (2) Preliminary screening of the literature based on the title, keywords and abstracts; (3) Adding the latest research progress and new references from the original literature; (4) Summarizing and organizing the existing literature.

## 2. Main Component Types and Activities of ZEO

### 2.1. Terpenoids and Activities

Terpenes are a class of compounds characterized by the isoprene unit (C_5_H_8_) as the basic structural unit [6], and their structural diversity mainly stems from the involvement of two key precursors, isopentenyl diphosphate (IPP) and dimethylallyl diphosphate (DMAPP) [7], a mechanism consistently reported in studies of terpenoid biosynthesis [8]. In the volatile oil of *Zanthoxylum bungeanum*, terpenoids represent the most abundant chemical group, with common constituents including limonene, β-laurene (10.009%), 3-bornene (6.512%), α-pinene, β-pinene, α-hydantoin, β-stigmasterol, sabinene, and anisole, etc. [9]. Notably, α-hydantoin and β-stigmasterol are not terpenoid derivatives; failure to exclude these compounds in comparative analyses may lead to inconsistencies in the terpenoid profile of ZEO, an oversight observed in several previous studies [10].

The composition and content of ZEO are affected by a variety of internal factors (e.g., plant genotype) and external factors (e.g., environmental conditions), but the magnitude of these effects varies among studies due to differences in experimental design. Devi et al. [11] specifically investigated seasonal variations in volatile oil components from different parts of *Zanthoxylum bungeanum* and found that decane in flowers and fruits was detectable only in spring and summer, whereas pinene content in leaves fluctuated markedly across seasons (4.36% in spring, 1.96% in summer, 6.09% in autumn, and 2.25% in winter); the authors attributed these changes to seasonal regulation of gene expression involved in terpenoid biosynthesis. However, a key limitation of the study by Singh et al. is the lack of control for geographical origin, which may confound the observed seasonal effects—especially as geographical variation is widely recognized as a major factor influencing the chemical composition of ZEO [12]. Tang et al. [13] compared the volatile oil compositions of *Zanthoxylum bungeanum* pericarps and non-pericarps tissues, and found that the dominant components in the main chemical classes were consistent, but their relative contents differed significantly. In their study, the main components of ZEO included limonene (18.267%), 4-terpineol (10.625%), laurin(10.009%), α-pinene (8.199%), pinene acetate (6.499%), 3-camphene (6.512%), and pinene (6.499%). The contents of d-d-limonene and sabinene in *Zanthoxylum bungeanum* seeds were 5.39% and 2.14% higher than those in non-*Zanthoxylum bungeanum* seeds, respectively. Trung et al. [14] complemented these findings by investigating geographical variation and reported distinct differences in the main components of volatile oils from different parts of Vietnamese *Zanthoxylum*: leaves were dominated by (E, E)-α-farnesene (19.6%), flowers by β-elemene (23.7%), and fruits by limonene (41.2%). Direct comparison between the results of Trung et al. [14] (Vietnamese *Zanthoxylum*) and Tang et al. [13] (Chinese *Zanthoxylum*) revealed a notable discrepancy in fruit limonene content (41.2% vs. 18.267%), which likely reflects differences in geographical origin and cultivar. Nevertheless, neither study standardized extraction methods—a critical methodological variable—making it difficult to distinguish the contributions of genetic and experimental factors to these discrepancies.

In terms of bioactivity, Khamtache-Abderrahim et al. confirmed that several terpenes present at low relative contents (e.g., γ-pinene, α-pinene) exhibited potent scavenging activity against DPPH and ABTS^+^ radicals, and suggested that their antioxidant capacity is related to methylene groups in their chemical structures [15]. Limonene, which is present at a relatively high level (18.267%) in ZEO, has been reported to exert multiple biological functions, including antibacterial, insecticidal, anti-inflammatory, and expectorant effects, as well as gallstone dissolution, and cancer cell growth inhibition. In addition, stilbene oxide, as an important component of the volatile oil of many Chinese herbs, possesses traditional medicinal properties such as soothing the liver and to relieve pain, lowering blood pressure and regulating menstruation, clearing heat and draining dampness, and inducing diuresis to reduce swelling [16]. The contents of terpenoids in ZEO are presented in Table 1.

### 2.2. Alcohol Compounds and Activity

Alcohols represent the second most abundant class of components in ZEO, following terpenes, with linalool, 4-terpineol, and eucalyptol as the predominant constituents. Several studies have analyzed their specific composition and relative contents. For instance, one investigation identified 28 alcohol derivatives, among which linalool (17.62 ± 0.40% ~ 23.89 ± 1.02%), eucalyptol (5.91 ± 0.02% ~ 7.46 ± 1.09%), α-pinitol (1.22 ± 0.09% ~ 1.94 ± 0.25%) and geraniol (1.11 ± 0.06% ~ 1.81 ± 0.03%) were the most abundant [31]. In another study, 11 alcohol compounds were detected across six *Zanthoxylum bungeanum* volatile oil samples, dominated by β-linalool (1.55 ± 0.02% ~ 21.17 ± 0.02%), 4-terpineol (1.08 ± 0.01% ~ 13.36 ± 0.03%), and eucalyptol (4.86 ± 0.01% ~ 9.17 ± 0.01%) [32]. Notably, the alcohol profile and relative contents in *Zanthoxylum bungeanum* essential oils are significantly affected by factors including pretreatment procedures and cultivar type, it consistent with previous findings that these variables contribute to the heterogeneity of ZEO components.

In terms of flavor contribution, alcohols play an important role in the formation of the overall aroma of ZEO. Niu et al. [33] found that eleven constituents, including linalool and 4-terpineol, in the essential oils of *Zanthoxylum bungeanum* corns from seven different origins collectively determined the flavor differences of the samples and influenced the overall flavor of the ZEO. Niu et al. further noted that the pungent, woody, and greenish aromas in the volatile oils of *Zanthoxylum bungeanum* corns were related to linalool and other compounds, while the sweet aroma was related to geraniol and others [33]. Liu et al. also confirmed that linalool was the major aroma contributor in green and red *Zanthoxylum bungeanum*, while 1,8-cineol (19%), γ-pinitol (16%), and geraniol (11%) also played important roles in the aroma of red *Zanthoxylum bungeanum* [34]. The above studies consistently showed that linalool and other alcohols play a central role in the flavor profile of ZEO. In terms of bitter flavor characteristics, the bitterness of ZEO is mainly derived from ketone and alcohol components. Although the bitter intensity of alcohols was weaker than that of ketones, high concentrations of alcohols still had a certain influence on the bitter flavor and exhibited a synergistic enhancement effect with ketones [35]. Among the many volatile alcohols, linalool is the most abundant. Linalool is a monoterpene alcohol that exists in two enantiomeric forms [36] and is widely used in the flavor and cosmetic industries for its unique aroma [37].

In terms of pharmacological activities, alcohols often act synergistically with other volatile components such as terpenes. For example, Liang et al. reported that linalool and limonene exert synergistic insecticidal activity [25], while Wang et al. found that linalool, limonene, and sabinene jointly alleviated the oxidative damage of myofibrillar proteins (MP) by malondialdehyde (MDA) [38]. In addition, linalool itself has a wide range of pharmacological activities, including antimicrobial, insecticidal, antidepressant, lipid-regulating, anti-adipogenic, analgesic, anti-inflammatory, anti-angiogenic, anticancer effects, as well as ameliorative effects against Alzheimer’s disease; it also displays antiviral and sedative properties, and inhibits the growth of bacteria such as *Escherichia coli* and *Staphylococcus* spp. [39]. Other alcohols, including α-terpineol and 4-terpineol, possess acaricidal activity [40]; 1,8-cineol, a major constituent in the volatile oil of various of traditional Chinese herbal medicines, shows hepatoprotective, antitussive, anti-ulcer, and anticoagulant activities [41]; nerolidol exhibits antitumor, antibacterial, and antiparasitic effects [42]; α-salicylenol possesses cooling and antipyretic activity [43]; cedrol combines insecticidal and platelet-activating factor (PAF) binding inhibitory activities [44]; and cedrol can suppress the proliferation and induce the apoptosis of breast cancer cells [45]. Notably, most of these pharmacological studies are based on in vitro experiments or animal models, and their translational value to human physiology remains to be verified. The contents of alcoholic compounds in ZEO are presented in Table 2.

### 2.3. Other Compounds (Aldehydes, Ketones, Esters, Etc.) and Activities

In addition to terpenoids and alcohols, ZEO also contains trace components of esters, ketones, aldehydes, and alkanes. Notably, the types and contents of these trace components can vary significantly depending on analytical approaches and sample characteristics, as demonstrated by two comparative studies: Wu et al. used headspace solid-phase microextraction coupled with gas chromatography–mass spectrometry (HS-SPME-GC-MS) to analyze the volatile oils from the pericarps and leaves of *Zanthoxylum bungeanum* collected from Sichuan and Shanxi provinces, and identified a total of 31 olefins, 14 alcohols, nine esters, three ketones, two aldehydes, one acid, one phenol, and one alkane [50]. In contrast, Zhang et al. [31] employed conventional GC-MS (without HS-SPME pretreatment) to analyze fresh and dried *Zanthoxylum bungeanum* samples; detecting a total of 114 major components—far more than those identified by Wu et al. [50]. These included 50 olefins (41.35 ± 7.68% ~ 61.50 ± 2.68%), 28 alcohols (32.51 ± 2.06%), 16 esters (0.26 ± 0.02% ~ 0.35 ± 0.08%), nine aldehydes (0.16 ± 0.05% ~ 0.24%), eight alkanes (0.07% ~ 0.12 ± 0.02%) and five ketones (0.86 ± 0.17% ~ 5.93 ± 3.79%) [31]. The observed discrepancies between the two studies are primarily attributed to differences in analytical techniques, sample pretreatment methods, and geographical origins—key factors that can significantly influence the composition and detection of ZEO trace components. A key limitation of these two studies lies in the inconsistencies in analytical methodologies and sample matrices: specifically, the HS-SPME-GC-MS technique used by Wu et al. has higher sensitivity for volatile trace components but may underestimate the content of non-volatile constituents; in contrast, the conventional GC-MS technique used by Zhang et al. can capture more non-volatile or semi-volatile components but has lower sensitivity for low-abundance volatile trace components [31,50].

Despite their low content, these trace components play a key role in the formation of the overall flavor of *Zanthoxylum bungeanum.* Specifically, several studies have identified the specific contributions of individual trace components to ZEO’s flavor profile: Wu et al. pointed out that bornyl acetate has a lemon and lavender-like aroma, while linalyl acetate presents a mild and sweet scent [50]. Building on this finding, Zheng et al. [24] further found that nerolidyl acetate and geranyl acetate contribute to the floral aroma of *Zanthoxylum bungeanum* pericarps; they also observed that the content of geranyl acetate increases with temperature, indicating that it is more sensitive to environmental changes, whereas nerolidol is less affected by climate. However, a key inconsistency and limitation of Zheng et al.’s study is that this temperature-dependent variation has not been validated in *Zanthoxylum bungeanum* samples from other geographical origins, a gap that severely limits the generalizability of this conclusion. In addition to these ester components, Zheng et al. confirmed that citronellal is a characteristic component in the essential oil of *Zanthoxylum bungeanum* and plays an important role in the formation of citrus-like aroma [24].

In terms of pharmacological activities, esters, aldehydes, and ketones also exhibit significant bioactivities. For example, as the dominant ester component in ZEO, bornyl acetate shows promising anti-aging effects [51]; however, it should be emphasized that this anti-aging effect has not been validated in animal or clinical models, and it is premature to extrapolate this to in vivo efficacy. Similarly, camphor, as another important trace component, possesses diverse activities including analgesic, anti-inflammatory, mosquito-repellent, and antifungal properties. Beyond these volatile components, ZEO is also rich in fatty acids, which account for the largest proportion (up to 53.303%), mainly including eicosapentaenoic acid (EPA), docosahexaenoic acid (DHA), and arachidonic acid (ARA, eicosatetraenoic acid). Owing to its mild operating temperature and unique physicochemical properties, supercritical CO_2_ extraction enables efficient retention of these high-molecular-weight fatty acid components. Notably, arachidonic acid and docosahexaenoic acid, as long-chain polyunsaturated fatty acids (LC-PUFAs), can modulate inflammatory response in vivo, thereby exerting vital roles in disease prevention. The contents of other types of compounds in ZEO are presented in Table 3.

## 3. Factors Affecting the Composition and Content of ZEO

**Figure 2:**
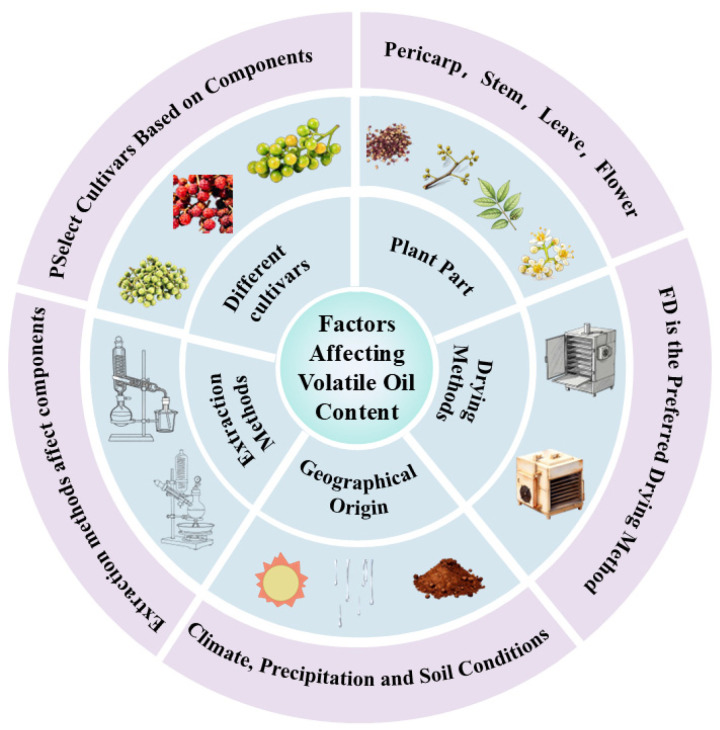
Factors influencing the composition and content of ZEO.

The composition and content of ZEO are not only closely related to the place of origin, but also affected by different parts, extraction methods and other factors, as depicted in Figure 2.

### 3.1. Different Parts of the Plant

Essential oils derived from *Zanthoxylum* species, including *Zanthoxylum bungeanum* essential oil (ZBEO) and *Zanthoxylum schinifolium* essential oil (ZSEO), can be extracted from a wide variety of plant parts, including the pericarp, stems, leaves, and flowers. There is growing evidence that the composition and content of these volatile oils vary significantly depending on the plant organ [62]. Existing studies consistently emphasize that fruit pericarps are particularly rich sources. One study reported that the essential oil content in the pericarp of the *Zanthoxylum bungeanum* was higher than that of the leaves, bark, and roots [56]. Another study further confirmed that the pericarp of *Zanthoxylum bungeanum* contains a more diverse array of volatile components and emits a stronger, more pungent odor than the leaves [50]. Collectively, these findings suggest that the pericarp may be the preferred organ for ZBEO extraction.

Notably, the distribution of specific compound classes appears to be organ-specific, which adds complexity to the selection of extraction organs. Xu et al. specifically noted that terpenes, a major class of volatile components in *Zanthoxylum* essential oils, are primarily synthesized in the oil cells of *Zanthoxylum bungeanum* leaves, suggesting that leaves may be regarded as the primary organ for terpene extraction [19]. In contrast, studies on mountain-grown *Zanthoxylum bungeanum* emphasize the importance of fruit and pericarp for other key constituents. A report identified linalool, D-limonene, and carvacrol as the main components of the essential oil from mountain *Zanthoxylum bungeanum* pericarps [58], a finding confirmed by Yuan et al., who demonstrated that linalool (74.16%) was the most abundant compound in the essential oil from mountain *Zanthoxylum bungeanum* fruits [49].

In conclusion, different parts of the *Zanthoxylum* species (i.e., *Zanthoxylum bungeanum* and *Z. schinifolium*) exhibit distinct chemical profiles in terms of essential oil composition and content. This organ-specific accumulation of volatile components implies that the rational selection of plant organs is crucial for the targeted extraction of specific essential oil compounds. To maximize resource utilization, future studies should focus on standardizing extraction protocols and addressing gaps in data from non-pericarp organs as well as inconsistencies in component analysis.

### 3.2. Different Varieties

The variety or species of *Zanthoxylum* is the fundamental determinant of its essential oil composition and content, with significant interspecific differences observed. This conclusion is supported by a growing body of comparative studies focusing on the essential oil profiles of various *Zanthoxylum* species, which consistently demonstrate the regulatory role of taxa in shaping terpenoid and other volatile component patterns [63]. For example, a study showed that the proportion of terpenes in *Z. schinifolium* (79.53%) was significantly higher than that of *Z. piperitum* A.P. DC. Consistently, the content of β-pinene in *Z. piperitum* A.P. DC (2.87%) was lower than that of *Z. schinifolium* (7.73%) [64]. It should be noted that this interspecific difference in terpenoid content is not an isolated phenomenon; Wu et al., in their research on *Zanthoxylum bungeanum*, further verified that variety exerts a significant influence on terpenoid component composition, which is consistent with the findings of the aforementioned study [50]. However, few studies have systematically compared the terpenoid biosynthetic pathways underlying these differences, which remains an unresolved challenge in clarifying the molecular mechanisms of chemotypic variation among *Zanthoxylum* species. In-depth comparative analyses have further revealed distinct chemical phenotypic profiles among different *Zanthoxylum* species, particularly in terms of their major volatile constituents. Yang et al. reported that linalyl acetate (15%) was the most abundant compound in *Zanthoxylum bungeanum*, whereas linalool (29%) was the major constituent in *Z. schinifolium;* Additionally, the contents of linalool and limonene in *Zanthoxylum bungeanum* were 16% and 2% higher than those in *Z. schinifolium*, respectively [56]. A limitation of this study, however, is that it only focused on two *Zanthoxylum* species and did not include other widely distributed taxa (e.g., *Z. armatum*), which may limit the generalizability of its conclusions to the entire genus. Ma et al. also demonstrated the effect of species on the volatile oils composition of *Zanthoxylum bungeanum* pericarp [65]. In addition, Sriwichai et al. found that *Z. armatum* has a unique aroma profile, exhibiting a citrus-floral specialized aroma type compared to other *Zanthoxylum* species [66].

In conclusion, the chemical composition and aromatic properties of essential oils from different *Zanthoxylum* species vary significantly, which is strongly supported by multiple peer-reviewed studies. This chemotypic variation is closely associated with the species/taxon of *Zanthoxylum*. It should be emphasized, however, that current conclusions are mainly based on comparative analyses of limited species and regions, and extrapolation to all *Zanthoxylum* taxa should thus be avoided. Accordingly, specific *Zanthoxylum* species can be targeted for different applications based on desired aromatic and functional compositions. Future studies should focus on filling the existing research gaps, such as systematically comparing the chemotypic variation of more *Zanthoxylum* species, clarifying the molecular mechanisms underlying these variations, and standardizing research methodologies to improve the reliability and comparability of research results.

### 3.3. Geographical Origin

Geographic origin is an important determinant of the chemical composition and aromatic properties of *Zanthoxylum bungeanum* plants, which is mainly attributed to regional differences in climate, precipitation and soil conditions [67]. This conclusion is supported by numerous studies focusing on the geographical variation of aromatic plants in the *Rutaceae* family, as environmental factors in different regions can directly regulate the synthesis and accumulation of secondary metabolites associated with aroma and chemical composition in plants [68]. For example, Wu et al. reported that the content of hydrocarbons and alcohols in *Zanthoxylum bungeanum* from northern China was lower than that in samples from central China. Similarly, mountain prickly ash (*Zanthoxylum armatum* DC.) from Sichuan and Yunnan exhibited significantly similar aroma profiles, which may be attributed to their similar longitude and thus comparable patterns of temperature, precipitation, and sunshine duration [50]. The effect of geography on specific compounds has been further demonstrated in studies of different cultivars. Xu et al. found that Youhuajiao (YHJ, an oil-type *Zanthoxylum bungeanum* cultivar) had the highest content of (+)-limonene; additionally, YHJ also had the highest content of α-pinene among the 11 leaf-use *Zanthoxylum bungeanum* cultivars [19]. However, it should be noted that the sample size of this study was limited to 11 leaf-use cultivars, and the conclusion may not be applicable to fruit-use cultivars—this is a potential limitation that needs to be considered in subsequent research [69]. In complementary studies, Zheng et al. noted that *Zanthoxylum bungeanum* pericarps from southwest and northwest China contain higher concentrations of limonene and linalool, which contribute to a more intense aroma [24]. This finding is consistent with the research results of Lan et al., further confirming that geographic environmental factors can regulate the accumulation of specific aromatic components in *Zanthoxylum bungeanum*. A study conducted in Hebei, China, identified terpinen-4-ol (18.42%), 1,8-eudesmol (15.49%), and limonene (7.47%) as the major components [70]. In contrast, a study from Sichuan, China, reported a distinctly different compositional profile, with D-limonene (15.17%), linalool (19.25%), and linalyl acetate (13.85%) as the major constituents [20]. The significant difference in essential oil (EO) compositions between the Hebei and Sichuan samples is mainly attributed to obvious regional differences in environmental factors between the two regions: Hebei province has a temperate monsoon climate, characterized by less precipitation, more sunshine hours, and neutral to alkaline soil, while Sichuan province has a subtropical humid monsoon climate, with abundant precipitation, moderate sunshine hours, and acidic to neutral soil. These environmental differences can regulate the activity of key enzymes involved in terpene compound synthesis (e.g., limonene synthase and linalool synthase), thereby affecting the accumulation of different terpene components in EOs [71]. Taken together, these findings indicate a consistent trend: the differences in chemical profiles observed in different production areas can be largely attributed to geographic differences in environmental factors such as soil composition, precipitation, and sunshine duration.

### 3.4. Drying Methods

The drying method used is a key factor affecting the composition and content of essential oils from *Zanthoxylum bungeanum*. Traditional techniques include sun drying (SD), hot air drying (HAD), far infrared drying (FID) and freeze drying (FD) [18,72]. In addition, emerging technologies such as radiofrequency-assisted hot air drying have been reported as an efficient and promising alternatives for the drying process [72]. The selected drying method exerts a significant effect on the final volatile component profile of EOs.

Zhao et al. demonstrated that Hanyuan *Zanthoxylum bungeanum* dried with hot air at 50 °C contained the highest levels of terpenes, esters, alcohols, and aldehydes, while sun-dried treated samples showed the opposite result [73]. This finding is consistent with those studies on other plant species. Suhata et al. reported that sun-dried (SD) and shade-dried (SSD) treated *Garcinia cambogia* samples had the lowest content of volatile constituents; in contrast, samples treated by forced-drying (FA), steam-heat-drying (SHD), oven drying (OD), and freeze-drying (FD) retained a higher level of volatile constituents [74]. However, it should be clarified that the superiority of different drying methods may vary with plant species, processing parameters, and target volatile components. Further supporting the superiority of advanced drying methods, a study has shown that the highest essential oil content was obtained from freeze-dried *Zanthoxylum bungeanum* Maxim., compared with samples treated by sun drying, hot air drying and far infrared drying [18].

In summary, empirical evidence strongly suggests that freeze drying (FD) is an efficient drying method for pretreating *Zanthoxylum bungeanum*, as it optimally preserves the yield and integrity of their essential oils, thus making it an ideal drying method for laboratory-scale sample pretreatment or high-value *Zanthoxylum bungeanum* products [12]. For large-scale industrial production, HAD (with optimized temperature parameters) may be a more cost-effective alternative, considering its balance between EO retention, processing efficiency, and cost. Future studies should focus on addressing unresolved challenges, such as optimizing drying parameters for different *Zanthoxylum bungeanum* varieties, developing low-cost and high-efficiency composite drying technologies, and clarifying the mechanisms by which drying methods affect the formation and retention of EOs [75].

### 3.5. Extraction Methods

Extraction methods play a vital role in determining the chemical composition and content of *Zanthoxylum bungeanum* (ZBEO) and *Zanthoxylum schinifolium* (ZSEO) essential oils. The commonly used techniques include hydrodistillation (HD) and supercritical CO_2_ extraction (SC-CO_2_) [76], which have been widely applied in the extraction of natural product essential oils due to their respective technical characteristics [77]. Systematic comparisons have demonstrated significant differences in the resulting volatile component profiles, depending on the extraction process. For example, relevant studies have shown that the linalool content extracted from *Z. schinifolium* by the HD method (32.54%) was significantly different from that obtained by the SC-CO_2_ method [49,58], suggesting that the choice of extraction method significantly affects the abundance of key constituents. Further supporting this, Lei et al. [52] reported that the main components of ZBEO extracted by the SC-CO_2_ method were olefins (41.372%), esters (35.870%), and alcohols (18.923%). This compositional pattern highlights how extraction techniques selectively influence the representation of different chemical classes in the final essential oil.

When comparing the two mainstream extraction methods for *Zanthoxylum* essential oils, HD is characterized by simple operation, low equipment cost, and mature technology, making it suitable for large-scale industrial production and rapid laboratory extraction [31]; however, its high extraction temperature may cause thermal degradation of heat-sensitive volatile components (such as some monoterpene alcohols and esters) in ZBEO and ZSEO, thereby leading to changes in chemical composition and even a decrease in biological activity [78]. In contrast, SC-CO_2_ uses low-temperature and non-toxic CO_2_ as the extraction medium, which can effectively retain heat-sensitive components and avoid solvent residues, thus better maintaining the natural chemical composition and biological activity of ZBEO and ZSEO [79]; nevertheless, this method has limitations such as high equipment investment, high operating cost, and relatively complex process control, which restricts its wide application in small and medium-sized laboratories and enterprises.

In conclusion, the extraction method is the major influencing factor for the differences in the volatile composition of *Zanthoxylum* essential oils (ZEOs). The selection of an appropriate extraction method should be comprehensively determined based on the research purpose (e.g., component separation, activity retention, or industrial production) and combined with the advantages and disadvantages of different methods.

## 4. Pharmacological Studies on the Therapeutic Properties of ZEO

### 4.1. Antimicrobial Activity

The antimicrobial effect of *Zanthoxylum bungeanum* pericarps has been utilized since ancient times. In traditional Chinese medicine (TCM), it has been widely used for treating sores, scabies and other dermatological conditions, for instance, the “Huajiao Hezhu Decoction” (containing “medical-grade” *Zanthoxylum bungeanum*) combines *Zanthoxylum bungeanum* with *Cornus officinalis* and *Cnidium monnieri* for decoction fumigation, which is specifically used for treating vaginal dampness and itching [80]; the “Hongshui Xuanzhu” incense-mustard medicine in TCM is applied for scabies and impetigo; in the National Chinese medicine prescriptions, the compound scabies ointment also combines *Zanthoxylum bungeanum* pericarps with *Andrographis paniculata* and sulfur, among other ingredients, for treating various types of intractable scabies and skin ulcers [81]. These traditional applications provide a historical and empirical basis for the modern exploration of the antimicrobial activities of *Zanthoxylum bungeanum* pericarps, though further clinical validation is required to translate traditional efficacy into evidence-based medicine [82].

Numerous studies have demonstrated that plant volatile oils possess broad-spectrum and low-toxicity antibacterial activities, and ZEO has also exhibited favorable antibacterial effects [83]. As a key antibacterial constituent in ZEO [84], linalool can exert its antibacterial effect by targeting the bacterial cell membrane. Its antibacterial mechanism mainly involves altering the structure and function of the cell membrane, increasing membrane permeability, inducing the leakage of intracellular contents and depletion of adenosine triphosphate (ATP); these events subsequently result in bacterial cell dysfunction and eventual death [85]. Notably, the antibacterial efficacy of linalool may vary depending on its concentration and the targeted bacterial species, a factor that should be taken into consideration when assessing its practical application potential [86].

Beyond the antibacterial activity of individual components, several experiments have confirmed the broad-spectrum antibacterial ability of ZEO itself. Studies have shown that it exerts a significant inhibitory effect on 10 Gram-positive bacterial strains and seven Gram-negative bacterial strains, including common Gram-positive bacteria such as *Staphylococcus aureus*, *Bacillus anthracis*, *Bacillus subtilis*, *Streptococcus pyogenes*, as well as potentially pathogenic Gram-negative bacteria such as *Vibrio cholerae* [87]. In terms of its mechanism of action, ZEO mainly blocks bacterial growth by impairing the integrity of microbial cell membranes and inhibiting spore germination; its overall antibacterial activity is usually superior to that of individual components (e.g., α-pinene) [88]. However, it should be emphasized that the antibacterial activity of ZEO can be affected by extraction methods and environmental factors such as temperature and pH, which limits the direct extrapolation of these findings to all practical application scenarios [89].

Khruengsai et al. reported that ZEO exhibited potent antibacterial activity against four common pathogenic bacteria, namely *Staphylococcus aureus*, *Staphylococcus epidermidis*, *Escherichia coli*, and *Pseudomonas aeruginosa* [90]. This finding is consistent with the broad-spectrum antibacterial property of ZEO observed in previous studies [54,55], yet it lacks a comparative analysis of the inhibitory efficacy among different bacterial strains, which is critical for clarifying the target specificity of ZEO. Another study indicated that blending ZEO with other essential oils produced synergistic antibacterial effects and enhanced the inhibition of all tested strains [91]. These observations suggest that ZEO has considerable potential as a natural alternative to clinical antibiotics for the management of bacterial infections, particularly in light of the worldwide concern over antimicrobial resistance. However, the clinical feasibility of such combined essential oil preparations remains to be verified via in vivo animal studies and clinical trials, since in vitro activity does not necessarily correlate with in vivo therapeutic efficacy [92]. Of note, although the antimicrobial properties of ZEO have been widely investigated, its application in food preservation has also garnered increasing interest, which is closely associated with its antimicrobial and antioxidant activities. For example, Wang et al. found that ZEO could prolong the induction period of lipid oxidation and delay the onset of microbial growth, thereby preserving the quality of rabbit meat patties [93].

**Figure 3:**
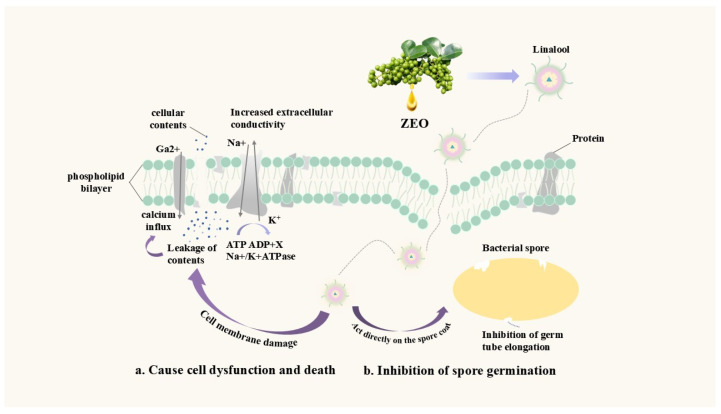
(**a**) ZEO induces cellular dysfunction and death: Linalool, a key component of ZEO, compromises the integrity of bacterial cell membranes, triggering the leakage of intracellular contents, elevated extracellular conductivity, and rapid ATP depletion, which collectively culminate in cell death. (**b**) ZEO inhibits spore germination: Linalool in ZEO exerts a direct effect on the outer coat of bacterial spores, blocking germination by suppressing germ tube elongation and ultimately leading to spore death.

Beyond its antibacterial and food preservation potential, ZEO has also been investigated for its regulatory effects on the intestinal microbiota, a property that expands its application scope in animal husbandry. Specifically, a study used 16S rRNA gene sequencing technology to investigate the modulatory effects of ZEO on the intestinal microbiota of ruminant animals [94]. The results demonstrated that ZEO could improve the structure and floral distribution of the small intestinal microbiota, thereby providing a theoretical foundation for the development of probiotics and microecological preparations for ruminants. From the perspective of the mechanism of action, ZEO exerts antibacterial effects mainly through two pathways. Its key component linalool can not only damage the integrity of bacterial cell membranes and trigger a series of reactions to induce cell apoptosis [53], but also act directly on the outer coat of bacterial spores to inhibit germ tube elongation and block germination [55], as detailed in Figure 3.

### 4.2. Antifungal

In traditional Chinese medicine, owing to its effects of drying dampness, killing insects, and relieving itching, *Zanthoxylum bungeanum* has been widely used in the treatment of fungal infectious diseases of the skin and mucous membranes caused by damp-heat accumulation or “insect venom” invasion. Its clinical application is mainly based on external therapy. A typical example is the Ginseng–Zanthoxylum decoction recorded in Yi Zong Jin Jian (The Golden Mirror of Medicine), in which *Zanthoxylum bungeanum* is contained with *Sophora flavescens*, *Phellodendron chinense*, and other medicinal materials, decocted and used for external washing to treat tinea, eczema, and other pruritic skin diseases, which demonstrates its efficacy in drying dampness and astringing sores [95]. In addition, folk prescriptions commonly include single-herb *Zanthoxylum bungeanum* or its combination with alum, vinegar, and other materials for decoction and external washing, which is applied to treat athlete’s foot (tinea pedis), tinea, and other diseases [96]. This traditional application is highly consistent with the antifungal effects of ZEO revealed by modern pharmacological research, verifying the scientific basis for the use of *Zanthoxylum bungeanum* essential oil in antifungal therapy in TCM from the perspective of modern pharmacology.

Modern pharmacological studies have demonstrated that ZEO not only possesses broad-spectrum antimicrobial activity but also exhibits significant inhibitory effects on a wide range of fungi, making it a promising natural plant-derived antifungal agent [97]. Studies have shown that ZEO has significant antifungal effects against *Penicillium* spp., *Aspergillus flavus*, and *Botrytis cinerea* [98,99]. However, it should be clarified that the antifungal activity of ZEO varies with the tested fungal strains and experimental conditions, a common limitation in current related research—most studies only focus on a single or a few fungal strains, lacking systematic verification across different fungal genera and species, which results in the need to improve the universality of research conclusions. Studies by different scholars have further confirmed the antifungal potential and characteristics of ZEO. Liao et al. reported that the minimum inhibitory concentration (MIC) of ZEO against *Malassezia* was 2.5 mg/mL and a minimum fungicidal concentration (MFC) was 10.0 mg/mL, suggesting that ZEO has potential application value as an antifungal control agent for this strain [30]. In contrast, Li et al. compared the antifungal activity and mechanism of ZEO and its main component α-pinene, and found that the inhibitory effect of ZEO was significantly stronger than that of α-pinene, with MICs of 6.25% and 12.50%, respectively [88]. Both exert antifungal effects by damaging the integrity of fungal cell membranes and inhibiting spore germination. The results of this study indicate that the antifungal activity of ZEO does not depend on a single active component, but is more likely a comprehensive effect of the synergistic action of multiple components.

At the level of research methodology, Liao et al. employed the mycelial growth inhibition method to evaluate the activity of ZEO against 15 plant pathogenic fungi, and confirmed that ZEO had a broad-spectrum inhibitory effect, among which the inhibitory effects on *Rhizoctonia solani* and *R. cerealis* were the most significants [30]. Although the mycelial growth inhibition method selected in this study has advantages such as simple operation, low cost, and intuitive observation of inhibitory effects, it also has obvious limitations—it cannot accurately reflect the inhibitory effect of ZEO on fungal spores or the dynamic changes in fungal growth [98]. Furthermore, Liao et al.’s study further expanded the application scenarios of ZEO; their findings demonstrated that ZEO has the potential to be applied in the postharvest preservation of fruits and vegetables [30].

In conclusion, ZEO possesses broad-spectrum and effective antifungal properties and shows inhibitory ability to a variety of plant and human pathogenic fungi, endowing it with significant value for the development of agricultural preservation technologies and the prevention and control of fungal disease. However, objectively speaking, there are still numerous limitations in current research on the antifungal activity of ZEO, mainly including inconsistent research results caused by differences in methodologies, insufficiently in-depth research on antifungal mechanisms, and a lack of verification in practical application scenarios. Therefore, future research should focus on these unresolved challenges and accelerate the industrial development and practical application of ZEO as a natural plant-derived antifungal agent by standardizing research methodologies, strengthening in vivo experiments and practical application studies, and further clarifying the antifungal mechanism of ZEO.

### 4.3. Anti-Inflammatory

In traditional Chinese medicine, due to its pungent flavor and warm nature, *Zanthoxylum bungeanum* has been widely used to treat inflammatory pain caused by cold and dampness, or stagnation of qi and blood stasis [100]. In classic TCM formulae, *Zanthoxylum bungeanum* is often used as a principal or assistant herb to relieve paralytic pain, cold pain in the stomach and abdomen, and toothache [22].

Although direct studies on the anti-inflammatory capacity of ZEO remain relatively limited, a growing body of evidence has shown its potential to regulate inflammatory responses in various inflammation-related disease models. Zhang et al. found in a dextran sulfate sodium (DSS)-induced mouse model of experimental colitis that ZEO significantly reduced the production of pro-inflammatory mediators; the underlying mechanism was related to the modulation of the NF-κB and PPARγ signaling pathways as well as the inhibition of NLRP3 inflammatory vesicle activation, suggesting that ZEO may be a dietary strategy to prevent ulcerative colitis [101]. Further studies showed that ZEO could reverse the LPS-induced imbalance between pro-inflammatory factors (TNF-α, IL-6, IL-1β) and anti-inflammatory factors (IL-10) in colonic epithelial cells, and downregulate the mRNA levels of inflammation-related genes (e.g., VCAM-1, TLR8, IL-1β, IL-11) in the colonic tissues [22]. It should be clarified that the anti-inflammatory effects of ZEO observed in colitis models cannot be directly extrapolated to other inflammatory diseases (e.g., rheumatoid arthritis, neuroinflammation), as there are significant differences in the inflammatory microenvironment and pathogenic mechanisms among different disease types [22]. In addition, Li et al. [102] reported that *Zanthoxylum bungeanum* seed oil (ZSO) reduced serum TNF-α, IL-1β, and IL-6 levels and exerted anti-inflammatory effects by regulating the phosphorylation of IκBα and NF-κB p65 in a rat model of burn injury. Xu Tangling et al., using a copper comb burn model, also confirmed that both high and low doses of ZSO could inhibit the elevation of inflammatory factor levels in rats and delay the necrosis of traumatic tissue [103]. However, these two burn-related studies have certain limitations; for example, the specific bioactive constituents in ZSO responsible for its anti-inflammatory activity remain unclear, and this issue needs to be addressed in future research.

In summary, ZEO and its related derivatives exert anti-inflammatory effects through multiple mechanisms, including inhibiting the release of pro-inflammatory factors, regulating the expression of anti-inflammatory factor, blocking the activation of NF-κB and other related signaling pathways, and inhibiting NLRP3 inflammasome activation [104], thus possessing the potential to become therapeutic agents for inflammatory diseases. These components have shown potential therapeutic value in inflammation-related diseases such as ulcerative colitis and burn-induced inflammation. However, existing studies still have certain limitations, including small sample sizes, insufficient evaluation of long-term efficacy, unclear specific bioactive constituents, and a lack of direct comparative studies with other plant-derived anti-inflammatory agents. Therefore, future research should focus on addressing these unresolved challenges, clarifying the structure–activity relationship of bioactive components derived from *Zanthoxylum bungeanum*, and conducting more in-depth mechanism studies to provide more solid experimental evidence for their clinical application in the treatment of inflammatory diseases [105].

### 4.4. Anti-Tumor

Beyond its anti-inflammatory properties, *Zanthoxylum bungeanum* and its essential oil also show promising antitumor potential, which has been gradually explored in modern cutting-edge research. In traditional Chinese medicine theory, although there are no clear disease records directly corresponding to the modern term “tumor”, *Zanthoxylum* is often used as a adjuvant medicine in modern compound research for resolving masses and relieving pain. Modern cutting-edge studies have provide solid scientific evidence for the antitumor potential of ZEO [106].

**Figure 4:**
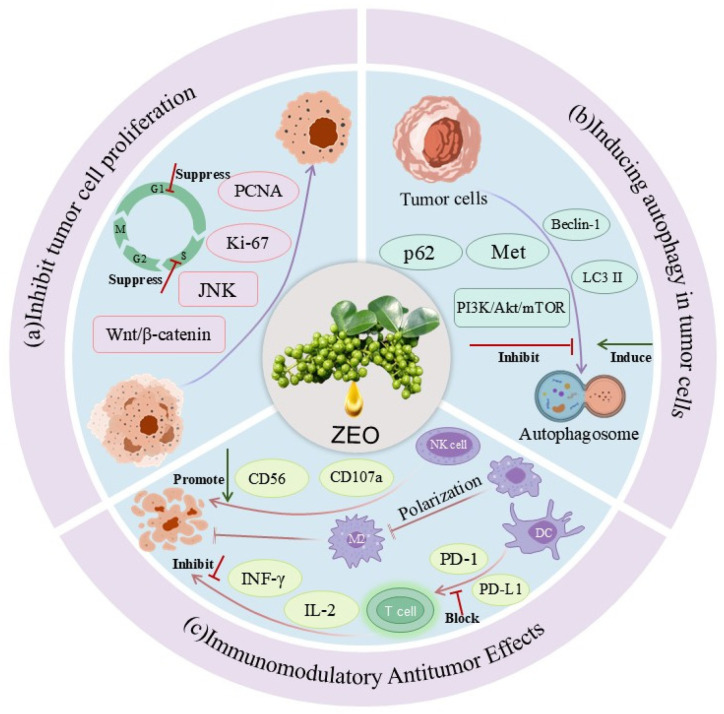
(**a**) Inhibition of tumor cell proliferation: Downregulates the expression of proliferation markers such as PCNA (Proliferating Cell Nuclear Antigen) and Ki-67, activates the JNK (c-Jun N-terminal Kinase) signaling pathway, inhibits the Wnt/β-catenin pathway, and induces cell cycle arrest. (**b**) Induction of tumor cell autophagy: Regulates the PI3K (Phosphatidylinositol 3-Kinase)/Akt (Protein Kinase B)/mTOR (Mammalian Target of Rapamycin) pathway, upregulates autophagy-related proteins including Beclin-1 and LC3 II (Microtubule-Associated Protein 1 Light Chain 3 II), and promotes autophagosome formation. (**c**) Immunomodulatory antitumor effect: Promotes the activation of NK (Natural Killer) cells and polarization of dendritic cells, enhances the ability of T cells to secrete IFN-γ (Interferon-γ) and IL-2 (Interleukin-2), and inhibits the PD-1 (Programmed Cell Death Protein 1)/PD-L1 (Programmed Cell Death Ligand 1) immune checkpoint pathway, thereby activating the antitumor immune response.

In recent years, it has been found that the active ingredients in ZEO have inhibitory effects on a variety of tumor cells [107]. Yuan Taining et al. found that low concentrations of ZEO induced the death of cervical cancer cells, while high concentrations directly killed cancer cells [108]. Han Shengnan et al. reported that ZEO exerted growth inhibitory effects on cervical cancer HeLa cells, lung cancer A549 cells, and leukemia K562 cells, indicating that it has a broad-spectrum antitumor potential in vitro [109]. However, it should be clarified that the antitumor activity observed in these in vitro studies is limited to specific cell lines, and the applicability of such effects to other tumor types remains to be further verified. Regarding the antitumor mechanisms of ZEO and its related products, different research teams have reported various regulatory effects on the cell cycle and cell death pathways. Pang et al. [110] showed that pressed *Zanthoxylum bungeanum* seed oil (ZSO) induced G1-phase arrest, prevented mitosis, and triggered apoptosis in human melanoma A375 cells. In contrast, Bai et al. [111] further found that ZSO can exert antiproliferative effects by causing S-phase arrest, reducing phosphorylation levels, and inducing autophagy in human laryngeal epidermoid carcinoma Hep-2 cells. The differences in the phases of cell cycle arrest in these two studies may be attributed to the differences in tumor cell types, which have unique genetic backgrounds and sensitivity to natural products [112]. This indicates that the antitumor mechanism of ZEO and its derivatives is cell-type dependent, and the specific regulatory factors still need to be further clarified through research. In addition to in vitro studies, in vivo animal experiments have further confirmed the antitumor potential of ZEO and its related products. In animal models, Wen Tingru et al. found that nebulized inhalation of supercritical CO_2_-extracted ZEO improved colonic mucosal lesions and reduced tumorigenesis using an AOM/DSS-induced mouse colorectal cancer model. The mechanism is related to the activation of the α-7nAChR receptor, regulation of cholinergic anti-inflammatory pathway, and down-regulation of IL-6 expression [113]. The mechanism is depicted in Figure 4.

In conclusion, ZEO, as a natural product derived from TCM, can inhibit tumor cell growth through cell cycle arrest, induction of autophagy and apoptosis, and exert an antitumor effect in vivo by regulating the immune and inflammatory microenvironment, endowing it with significant research value for further development as a natural antitumor agent. However, existing studies still have certain limitations: (1) most in vitro studies focus on a few tumor cell lines, and its broad-spectrum antitumor activity still needs to be verified in more types of tumor cells; (2) the in vivo studies have small sample sizes and short observation periods, and its long-term antitumor efficacy and safety are not yet clear; (3) the specific active components in ZEO that exert antitumor effects have not been fully clarified, and its structure–activity relationship still needs further exploration [114]. Despite these unresolved challenges, ZEO still has important research value as a potential natural antitumor drug, and further in-depth studies are needed to promote its clinical transformation and application.

### 4.5. Insecticidal and Antipruritic Effects

In classic prescription, *Zanthoxylum bungeanum* is the key TCM herb for expelling intestinal worms, with particular efficacy in killing roundworms (*Ascaris lumbricoides*) [115]. The most representative formula reflecting its anthelmintic activity is Wumei Pill, recorded in Treatise on Febrile Diseases (Shang Han Lun), which can effectively soothe roundworms and alleviate worm-induced pain. This formula has long been honored as the “grandfather formula” for roundworm expulsion in TCM. In addition, *Zanthoxylum bungeanum* is also used for worm accumulation complicated by cold pathogen (combined with dried ginger, e.g., An Ascaris Lizhong Tang) or cases requiring both anthelmintic and spleen-invigorating effects (combined with Shenqu, e.g., Fei’er Shachong Wan [Fat Children’s Anthelmintic Pill]). Externally, it can be formulated with alum (e.g., Xi Wen Fang [Tattoo Wash Formula]) for the treatment of *Trichomonas vaginalis* [115], skin parasitic infestations and dental caries [116]. These traditional applications provide a historical and clinical basis for modern research on the insecticidal and antipruritic effects of *Zanthoxylum bungeanum*, but further evidence from contemporary pharmacological studies is needed to verify their underlying mechanisms of action.

Modern research has demonstrated that the insecticidal mechanism of *Zanthoxylum bungeanum* is mainly attributed to its volatile oil, which exerts a paralyzing effect on the parasitic nervous system to achieve insecticidal effects [25]. Specifically, ZEO is rich in various of components that exhibit repellent, oviposition-inhibitory, fumigant, and contact toxic activities against a variety of pests, collectively endowing it with significant insecticidal and antipruritic efficacy [117]. In terms of insecticidal effects, studies have shown that green *Zanthoxylum bungeanum* extracts exert significant repellent and toxic effects on peach aphid and radish aphid, as well as inhibiting their growth and development [118]. However, it should be noted that, compared with synthetic insecticides, the insecticidal activity of these extracts is relatively moderate, and their stability under field conditions still requires further verification [119]. Researchers such as Zhao et al. [120] investigated the optimal insecticidal conditions of the essential oil of green *Zanthoxylum bungeanum* in Northeast China, setting the fumigation duration of 24 h, 48 h, and 72 h as the three key time points, with a series of different concentration gradients established for each time point. Liang, J.Y. et al. demonstrated that the essential oil of *Zanthoxylum bungeanum* exhibited significant acute toxicity and promising insecticidal activity against two stored-product insects, namely *Tribolium castaneum* (adults and larvae) and *Lasioderma serricorne* (larvae), and notable synergistic interactions were observed between its major components, limonene and linalool [25]. This finding is consistent with the results of other plant essential oil studies, which have also reported concentration- and time-dependent fumigant toxicity against pests [121]; however, the potential molecular mechanism by which ZEO induces neural paralysis in parasites has not been fully elucidated, which is a key limitation of current research. In terms of antipruritic effects, *Zanthoxylum bungeanum* is traditionally used for warming the middle energizer, dispersing cold, eliminating dampness and relieving itching. From a modern pharmacological perspective, Luo et al. demonstrated, using a rat skin pruritus model, that *Zanthoxylum bungeanum* essential oil (ZEO) exhibits a significant alleviative effect on acute pruritus [122].

In conclusion, ZEO can be used as a green pesticide to reduce chemical pesticide residues and ensure agricultural product safety, and also has the potential to be developed into a natural anti-mite and antipruritic topical preparation in the pharmaceutical field. However, current research still has several limitations: (1) the potential molecular mechanisms underlying the insecticidal and antipruritic activities of ZEO remain unclear; (2) most studies are in vitro experiments, with insufficient in vivo validation and field trials; (3) the stability and bioavailability of ZEO still need to be improved to meet the requirements of practical applications. Future studies should focus on solving these unresolved problems, clarify the structure–activity relationship of bioactive components in ZEO, and optimize its extraction and formulation processes to accelerate its industrialization and clinical application.

## 5. Preparation Technology and Application Areas of ZEO

**Figure 5:**
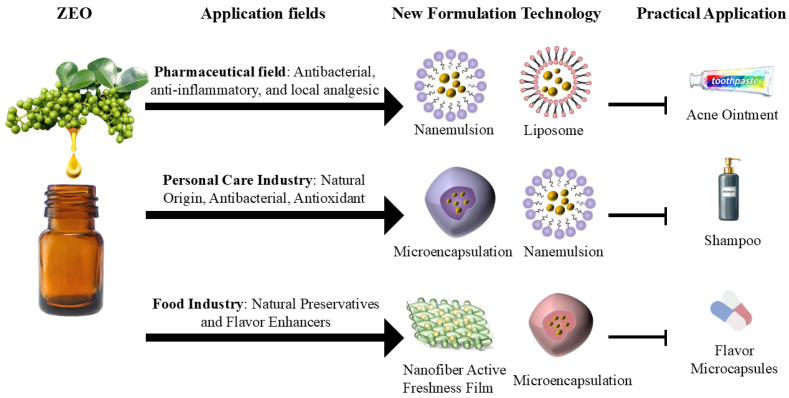
Schematic diagram of ZEO applications in pharmaceutical, daily chemical, and food industries.

ZEO possesses a broad-spectrum antimicrobial and antioxidant activities, as well as a unique flavor; however, its direct application is limited by inherent characteristics such as high volatility, poor water solubility, and chemical instability. In recent years, breakthroughs in novel formulation technologies including microencapsulation, nanoemulsions, liposomes, and composite films have significantly expanded the scope and depth of ZEO application in pharmaceuticals, cosmetics, and foodstuffs by improving its stability, controlling its release behavior, and enhancing its targeting ability. Schematic diagram illustrating the applications of ZEO in the pharmaceutical, daily chemical, and food industries are shown in Figure 5.

### 5.1. Pharmaceutical Applications

ZEO has shown promising applications in the pharmaceutical field, mainly based on its proven antibacterial, anti-inflammatory, and local analgesic bioactivities. Formulation studies have focused on overcoming its physicochemical limitations to achieve controlled release and targeted delivery of active ingredients.

In the development of topical antimicrobial and anti-inflammatory preparations, ZEO has a significant inhibitory effect on common dermatopathogenic bacteria such as *Propionibacterium acnes* (*Cutibacterium acnes*) and *Staphylococcus aureus* [123,124]. Traditional tinctures and ointments often suffer from poor skin permeability and easy inactivation of active ingredients. For this reason, technologies such as nanoemulsions and liposomes have been widely used [125]. For example, a study encapsulated ZEO in phospholipid-based liposomes, which not only significantly improved its transdermal permeability (especially for the stratum corneum), but also achieved follicle-targeted delivery, thus enhancing the therapeutic effect on acne and reducing skin irritation [126]. In addition, a drug-carrying hydrogel system based on chitosan-gelatin has been developed for wound dressing, which can continuously release the antimicrobial components of ZEO and achieve synergistic therapeutic effects with the hemostatic and wound-healing properties of chitosan [127].

In oral drug delivery systems, ZEO is often used as a natural odorant to improve drug palatability, as its inherent pungent odor easily leads to poor medication adherence in patients. Microencapsulation of ZEO via spray drying, with maltodextrin/gum arabic as the wall material can effectively mask its pungent and irritating odor, thus improving patients’ medication adherence [128]. In addition, cutting-edge studies have explored its preparation as a β-cyclodextrin inclusion complex and its application in colon-targeted drug delivery systems, which can regulate the release of ZEO through colonic flora-specific enzymes to realize its potential role in anti-intestinal inflammation [129]. Compared with traditional oral formulations, this colon-targeted delivery system can increase the local concentration of ZEO in the colon, reduce systemic side effects, and enhance its therapeutic effect on intestinal inflammatory diseases [130].

### 5.2. Applications in the Field of Daily Chemicals

In daily chemical products, ZEO is of great interest due to its natural origin and antimicrobial and antioxidant properties [131]. Formulations research in this field focus on addressing the challenges imposed by its high volatility, poor stability, and strongly irritating odor [132]. These inherent drawbacks of ZEO have restricted its widespread application in daily chemical products; therefore, targeted modification and formulation optimization technologies are urgently needed to overcome these limitations. In efficacious personal care products, ZEO has been used in the development of anti-dandruff and anti-itch shampoos and body washes [133]. The main active components of ZEO (e.g., linalool, limonene) effectively inhibit *Malassezia* [30], thereby relieving dandruff. Specifically, these terpenoid components can disrupt the cell membrane integrity of *Malassezia*, inhibit its metabolic activity, and thus alleviate dandruff and reduce scalp pruritus. However, direct incorporation of the active ingredients may lead to rapid loss during washing due to high volatility and poor water solubility, which significantly reduces the bioavailability and sustained efficacy of ZEO [134].

To tackle this problem, microencapsulation has been widely employed as an effective strategy to improve the performance of ZEO in personal care products [135]. For instance, microencapsulation of ZEO in wall materials such as sodium octenylsuccinate enables friction-triggered release during shampooing and long-lasting fragrance retention after washing, which greatly enhances product experience and efficacy durability [136]. Compared with other wall materials such as cyclodextrin and chitosan, sodium octenylsuccinate exhibits better compatibility with daily chemical formulations, lower toxicity, and more stable encapsulation efficiency, making it the preferred wall material for ZEO microencapsulation in personal care products. This technology not only improves user experience but also prolongs the efficacy of ZEO, providing a feasible strategy for its efficient application in personal care products.

In the application of sustained-release fragrances and natural preservatives, the unique “citrus-woody” aroma of ZEO renders it suitable for high-end perfumes and air fresheners [137]. Nanofiber membranes loaded with ZEO prepared via electrospinning can be used as sustained-release solid aromatherapy materials, with a release cycle several times longer that of traditional products [138]. Meanwhile, benefiting from its broad-spectrum antimicrobial activity, ZEO nanoemulsions have been applied as natural preservative system in cosmetics to partially replace the controversial parabens and meet the market demand for “additive-free” products. However, the high cost of electrospinning equipment and the poor mechanical properties of nanofiber membranes still limit large-scale industrial application; further studies are required to optimize the preparation process and reduce production costs.

### 5.3. Applications in the Food Sector

In the food industry, ZEO is mainly used as a natural preservative and flavor enhancer. Relevant formulation technologies aim to improve its dispersion, thermal stability, and oral bioavailability in complex food matrices [137]. Specifically, ZEO has the disadvantages of poor water solubility and insufficient thermal stability under food processing conditions, which have long restricted its practical application; thus, the development of nano-formulation technologies has become an effective approach to address thesee bottlenecks [139].

In the field of fruit and vegetable preservation, the traditional spraying method has technical limitations such as short duration of action and uneven distribution of active ingredients. Current research focuses on the developing preservation materials based on novel formulation technologies, such as edible coating films and controlled-release preservation pads [140]. For example, Li et al. successfully prepared polyvinyl alcohol/β-cyclodextrin nanofiber active packaging films loaded with ZEO using electrospinning technology. It was confirmed that strawberries and cherries treated with this active packaging film maintained good freshness during the 10-day storage period, whereas the untreated control group showed severe mold growth. This result indicates that the controlled-release system based on a nanofiber membrane can achieve continuous and uniform release of the active components in ZEO, which can effectively extend the shelf life of fruits and vegetables, and open up a new way to solve the technical limitations of the traditional preservation methods [141]. In addition, ZEO was prepared into plant-derived composite preservation microcapsules via the complex coacervation method by combining ZEO with cumin and garlic essential oil, and fixed on non-woven fabrics to prepare preservation pads. These pads can slowly release antimicrobial molecules during cold-chain logistics to achieve long-term protection of perishable foods such as blueberries and freshly cut vegetables.

Flavor customization and stabilization are key directions in meat processing and preservation. The flavor profile of ZEO obtained via supercritical CO_2_ extraction is closer to the characteristics of the raw material [142]. Through microencapsulation (with β-cyclodextrin and maltodextrin compounded as wall material) treatment, heat-resistant flavor microcapsules dedicated to meat products can be prepared and applied to sausages, dried meat and other products, effectively solving the technical problem of flavor loss during processing [143]. Meanwhile, replacing chemical preservatives is also a research hotspots: an antimicrobial emulsion prepared from ZEO and *Streptococcus lactis* was used for spray treatment on the surface of chilled meat, which can significantly inhibit the growth of pathogens, such as *Listeria monocytogenes* and achieve effects comparable to those of chemical preservatives, while being more in line with the “clean label” consumer trend [144]. However, compared with chemical preservatives, ZEO has a higher cost and the emulsion has a shorter shelf life, which limits its large-scale industrial application; further optimization of formulation and production technologies is required.

The innovation of formulation technology is the core driving force for promoting the transformation of ZEO from basic research to industrial application. Cutting-edge technologies such as microencapsulation, nanocarrier systems, and edible composite membranes have significantly expanded the application of ZEO in high-value-added fields (e.g., pharmaceuticals, daily chemicals, and food) by effectively addressing the bottlenecks related to stability, solubility, release control, and sensory acceptance. Future research should focus on the following directions: (1) developing intelligent responsive formulations that can respond to specific environmental stimuli (e.g., enzymes or temperature) to achieve on-demand precise release of ZEO; (2) conducting in-depth studies on the synergistic effects of ZEO with other natural active ingredients or technologies to construct synergistic composite functional formulations; (3) systematically evaluating the metabolic kinetics and long-term biosafety of novel ZEO formulations in vivo, so as to provide solid data support for their compliant application in the fields of medicine and food.

## 6. Conclusions and Future Perspectives

### 6.1. Conclusions

Research on ZEO fully embodies the profound integration of traditional medicinal wisdom and modern scientific investigation. This review systematically clarifies that, as the primary active component of *Zanthoxylum bungeanum* Maxim. (Sichuan prickly ash), ZEO is not a single substance with a fixed composition, but a complex and tunable active system precisely regulated by multiple factors including cultivar, geographic origin, and processing techniques. Consistent with the characteristics of plant essential oils, the compositional complexity and variability of ZEO are closely associated with its ecological adaptation and medicinal potential, which is a key basis for its multi-target pharmacological activities.

ZEO is primarily composed of terpenoids and alcoholic compounds, which collectively contribute to its multidimensional pharmacological activities, including broad-spectrum antibacterial, anti-inflammatory, anti-tumor, and neuromodulatory effects. Modern scientific research has clearly elucidated the traditional functions of “warming the Middle Jiao to alleviate pain and relieve itching” through multiple clear molecular mechanisms, such as disrupting the structural integrity of microbial cell membranes, inducing apoptosis of abnormal cells. Furthermore, by revealing the synergistic regulatory effects of its active component group on multiple biological targets (including immune microenvironment homeostasis and ion channel function), this review provides an in-depth analysis of the modern scientific essence of the holistic therapeutic model of TCM—multi-component and multi-target actions. This precisely expounds the theoretical core of TCM concepts of pharmacological symbiosis and synergistic effects via formula compatibility. These findings systematically elevate the understanding of ZEO from apparent efficacy to analyzable, predictable molecular network regulation, laying a solid theoretical and technical foundation for its precise application in modern medicine and health products.

### 6.2. Future Perspectives

Moving forward, it is urgent to establish a cross-disciplinary collaborative innovation system that deeply integrates the core tenets of traditional Chinese medicine theory, modern technological methods, and rigorous translational science. This system aims to bridge the “translation gap” between laboratory findings and industrial applications. In the Future, the ZEO research will focus on three interrelated, logically progressive directions.

#### 6.2.1. Deepening Traditional Chinese Medicine Combination Theory

Rather than limiting investigations to the individual activity of ZEO, future research should prioritize unpacking the core essence of TCM compatibility theory—drawing on classical TCM formulas and following the sovereign, minister, assistant, and messenger (Jun–Chen–Zuo–Shi) formulation principle. By scientifically pairing ZEO with other plant essential oils with verified synergistic effects, a range of compound essential oil products can be developed for health maintenance, disease prevention, and adjunctive therapy, to optimize inter-oil synergies and improve overall efficacy. Additionally, advanced technologies such as computational systems pharmacology, multi-omics analysis, and organoid co-culture models should be integrated to quantitatively characterize the dynamic interaction networks between ZEO and key active components of classical TCM drug pairs (e.g., Sichuan *Zanthoxylum bungeanum*-dried ginger combinations). This approach will translate tacit, experience-based formulation knowledge into visualized, computable design frameworks. Ultimately, utilizing this research framework, the focus should shift towards next-generation compound essential oils that target specific pathological pathways (e.g., excessive inflammatory responses, immunosuppressive microenvironments), thus shifting formulation design from empirical combination to rational, evidence-based design, and providing theoretical support for the scientific development and utilization of TCM compound essential oils.

#### 6.2.2. Application of Modern Novel Formulation Technologies

To address the inherent application bottlenecks of ZEO—high volatility, poor stability, and low in vivo delivery efficiency, modern formulation technologies should be leveraged to enhance its stability, bioavailability, and targeting capability. For example, nanotechnology can be utilized to create ZEO-based nanocrystals or nanoemulsions, thereby improving its water solubility and bioavailability. Additionally, stimulus-responsive smart microcapsules, such as pH-responsive systems, can be engineered to enable precise, site-specific drug release in the gastrointestinal tract or at tumor loci. Furthermore, transdermal delivery systems based on liposomes or oleosomes can be developed to enhance skin penetration for the treatment of deep fungal infections or inflammatory pain. The integration of these novel formulation technologies will open up new, targeted avenues for ZEO applications in pharmaceutical care, the food industry, and daily chemical products.

#### 6.2.3. Application Transformation and Industrial Standardization

The translation of ZEO research into tangible market value relies on clear application scenarios and reliable product quality standards. Ultimately, the simultaneous advancement of application transformation research and the establishment of a comprehensive industry-standard system are imperative. Transformation research should focus on targeted product development validated by pharmacological activities. For instance, exploiting its anti-inflammatory and analgesic properties for medical device-type topical patches or harnessing its broad-spectrum antibacterial activity for natural preservatives or pet repellents. Simultaneously, a comprehensive quality standards system spanning the entire ZEO industrial chain needs to be implemented. Beginning from cultivation origins, standardizing processes such as variety selection, growing conditions, and cultivation management is crucial to ensure the consistency and stability of raw materials. For harvesting and processing, standardized operational protocols for timing, drying methods, extraction techniques, and other procedures are essential to uphold the content and quality of ZEO’s active components. Establishing comprehensive quality testing standards that employ modern analytical techniques, such as HPLC and GC-MS, as core quality control methods is vital. Developing characteristic fingerprint spectra and quantitative standards for key components, while strictly conducting safety assessments for heavy metals, pesticide residues, and microbial limits, ensures product quality compliance with both domestic and international regulations. This will lay a solid foundation for the industrialization of ZEO products.

In summary, current academic research on ZEO is undergoing a pivotal transition from traditional empirical paradigms to evidence-based, innovative research models. We must adhere to the principle of “preserving TCM essence while driving innovation” thoroughly exploring the traditional Chinese medical wisdom it encompasses. By integrating modern scientific and technological advancements, we can undertake systematic innovative research and promote its translational application. This will not only advance the modernization of TCM, but also foster the development of innovative, safe, and effective products across multiple fields, making meaningful contributions to human health and the growth of related industries.

## Figures and Tables

**Table 1:** The contents of terpenoids in ZEO and their extraction parts.

Compound	Molecular Formula	Structural Formula	Content	Extraction Method	Extract Parts	References
α-Thujene	C_10_H_16_	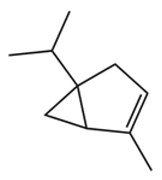	0.38–1.92	HD	pericarp, fruit	[17,18]
Sabinene	C_10_H_16_	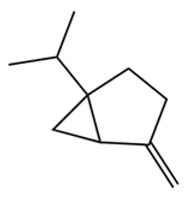	1.31–10.27	HD	leave, pericarp	[17,19]
β-Myrcene	C_10_H_16_	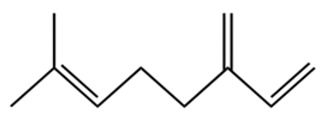	3.35–10.56	HD	fruit, pericarp	[17,20]
4-Carene	C_10_H_16_	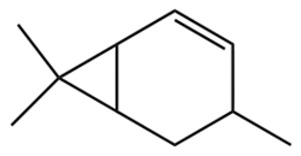	0.14–5.51	SFE, HD	pericarp, fruit	[18,21]
3-Carene	C_10_H_16_	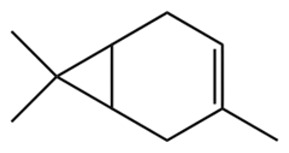	1.33–1.52	SD, HD	fruit	[22,23]
α-Phellandrene	C_10_H_16_	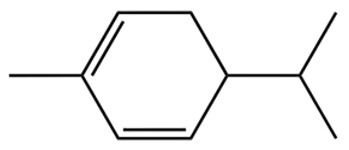	0.12–2.34	HD	pericarp	[17,21]
D-Limonene	C_10_H_16_	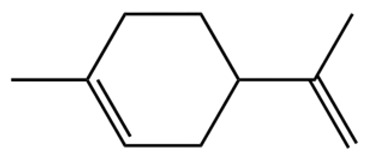	14.81–38.27	SD, HD	fruit	[18,22]
(E)-β-Ocimene	C_10_H_16_	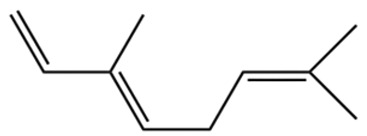	0.33–5.60	HS-SPME, HD	Pericarp, fruit	[18,24]
γ-Terpinene	C_10_H_16_	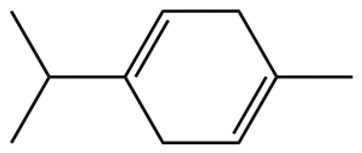	0.10–6.73	HD	leave, fruit	[18,19]
β-Terpinene	C_10_H_16_	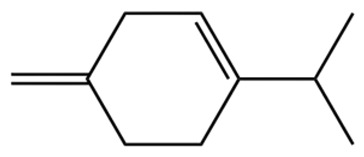	0.16–1.13	HD	aerial part	[23,25]
α-Caryophyllene	C_15_H_24_	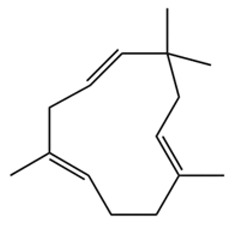	0.15–1.98	HD, HS-SPME	pericarp	[17,26]
β-Cadinene	C_15_H_24_	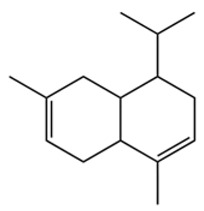	0.27–2.62	HD, SFE	aerial part, pericarp	[25,27]
γ-Cadinene	C_15_H_24_	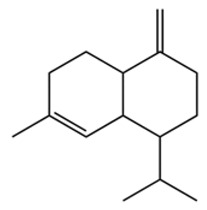	0.26–1.19	HD, HS-SPME	pericarp	[17,28]
α-Muurolene	C_15_H_24_	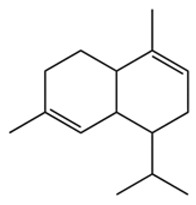	0.12–0.99	HD	fruit	[20,22]
β-Copaene	C_15_H_24_	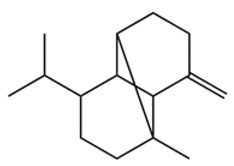	0.35–2.47	SC-CO_2_, SD	pericarp, fruit	[22,27]
o-Cymene	C_10_H_14_	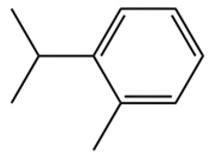	0.22–2.82	HD	fruit, leave	[18,19]
Cyclohexene	C_6_H_10_	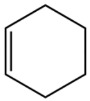	1.48–1.87	HD	fruit	[20,29]
Pseudolimonene	C_10_H_16_	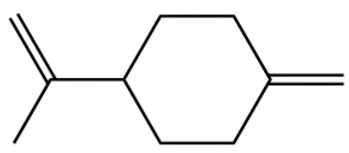	0.11–12.16	HD	fruit	[23,30]
β-Guaiene	C_15_H_24_	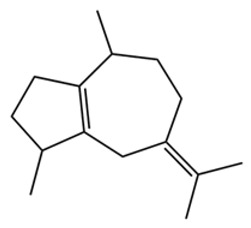	0.25–1.43	SD, HD	fruit	[22,23]
Naphthalene	C_10_H_8_	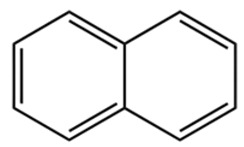	0.17–0.90	HD, SD	fruit	[20,22]

Note: If only one extraction method is listed, it indicates that the same method was used in both references. If two different methods are shown, the former corresponds to the method associated with the lower content, while the latter corresponds to the method associated with the higher content. If only one extraction part is listed, it indicates that the same part was used in both references. If two different parts are shown, the former corresponds to the part associated with the lower content, while the latter corresponds to the part associated with the higher content. HD = Hydrodistillation, SD = Steam Distillation, SFE = Supercritical Fluid Extraction, SC-CO_2_ = Supercritical CO_2_, HS-SPME = Headspace Solid-Phase.

**Table 2:** Contents and extraction methods of alcoholic compounds in ZEO.

Compound	Molecular Formula	Structural Formula	Content	Extraction Method	Extract Parts	References
L-alpha-Terpineol	C_10_H_18_O	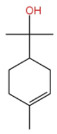	3.88–5.88	HD	fruit	[20,23]
4-Terpineol	C_10_H_18_O	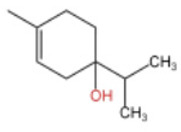	0.18–13.13	HS-SPME, HD	branch, pericarp	[24,46]
β-Eudesmol	C_15_H_26_O	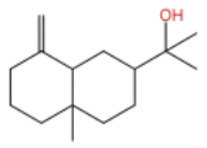	0.48–4.43	HD, SFE	leave, fruit	[19,47]
2-Cyclohexen-1-ol	C_6_H_10_O	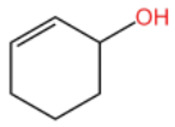	0.17–0.28	HD	fruit	[20,29]
Carveol	C_10_H_16_O	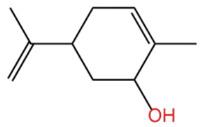	0.10–0.29	HD, SD	fruit	[22,48]
Nerolidol	C_15_H_26_O	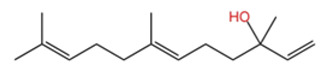	0.13–1.90	HD	fruit	[20,30]
2-Naphthalenemethanol	C_11_H_10_O	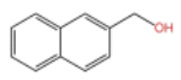	0.10–0.29	HD, SD	fruit	[20,22]
(+)-Citronellal	C_10_H_18_O	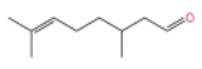	0.11–0.25	SC-CO_2_,HD	pericarp, fruit	[30,49]
Neral	C_10_H_16_O	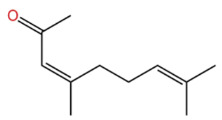	1.08–1.64	HD	aerial part, pericarp	[21,25]
Sabinene hydrate	C_10_H_18_O	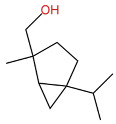	0.22–2.06	HD	pericarp, fruit	[17,30]

Note: Same as Table 1.

**Table 3:** The contents of other types of compounds in ZEO, extraction methods, and extraction parts.

Compound	Molecular Formula	Structural Formula	Content	Extraction Method	Extract Parts	References
Esters						
Linalyl anthranilate	C_17_H_23_NO_2_	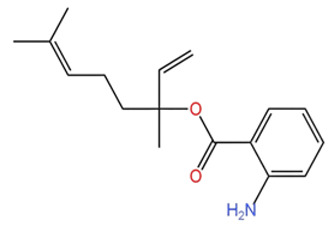	10.87–12.22	HD	aerial part, pericarp	[21,25]
α-Terpinyl acetate	C_12_H_20_O_2_	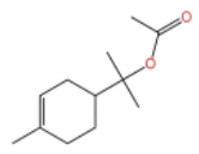	0.71–3.74	SC-CO_2_, SFE	pericarp	[21,27]
Terpinyl acetate	C_12_H_20_O_2_	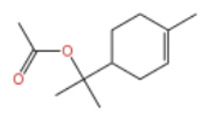	1.60–9.42	HD	pericarp, leaf	[17,19]
9,12-Octadecadienoic acid, ethyl ester	C_20_H_36_O_2_	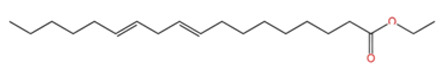	0.60–0.86	SC-CO_2_	fruit	[48,52]
Nerol acetate	C_12_H_20_O_2_	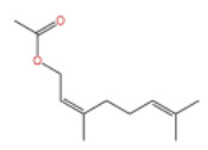	0.17–2.18	HS-SPME, HD	pericarp, aerial part	[24,25]
Sabinene hydrate	C_10_H_18_O	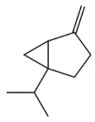	0.22–2.06	HD	pericarp, fruit	[17,30]
Acids						
Palmitic acid	C_16_H_32_O_2_	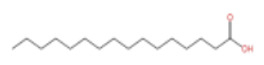	6.89–19.86	SFE, HD	fruit, seed	[47,53]
Myristic acid	C_14_H_28_O_2_	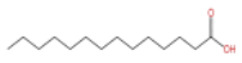	0.18–1.95	HD	leaves	[19,54]
α-Linolenic acid	C_18_H_30_O_2_	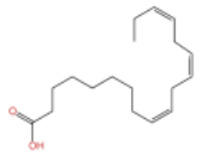	2.58–7.96	HD	leaves	[19,55]
Oxide						
Caryophyllene oxide	C_15_H_24_O	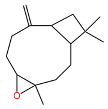	0.10–3.14	HS-SPME, HD	leaf	[19,28]
Trans-linalool oxide	C_10_H_18_O_2_	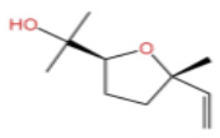	0.31–0.21	HD	pericarp	[56,57]
Ethers						
1,8-Cineole	C_10_H_18_O	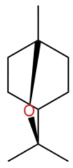	1.05–15.18	HD, HS-SPME	pericarp	[28,58]
4-Allylanisole	C_10_H_12_O	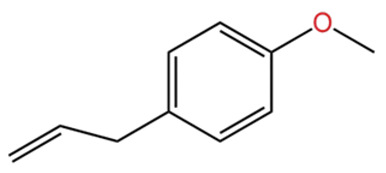	0.62–0.31	HD, UNE-HS-SDME	pericarp	[59,60]
Estragole	C_10_H_12_O	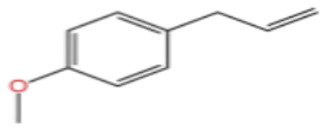	42.01–84.88	HD	seed, fruit	[23,53]
Ketones						
D-Carvone	C_10_H_14_O	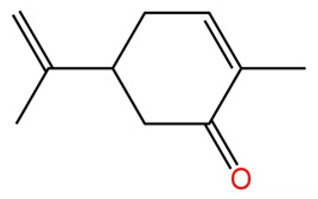	0.17–0.55	HD, SD	fruit	[20,22]
Xanthoxylin	C_10_H_12_O_4_	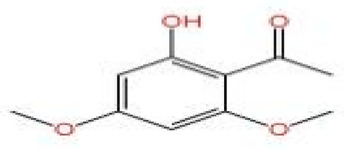	14.77–16.25	HD	pericarp, fruit	[23,61]

Note: Same as Table 1, with the addition of UNE-HS-SDME (Ultrasound-assisted Headspace Single-drop Microextraction).

## Data Availability

No new data were created or analyzed in this study. Data sharing is not applicable to this article.

## References

[1] Qu M., Liu Y., Wu H., Wang D. (2024). Research Progress on Extraction Method, Component Analysis, Bioactivity and Practical Application of *Zanthoxylum bungeanum* Maxim Essential Oil. China Condiment.

[2] Liu F., Pan H., Me G., Chen L., Chen H., Liu Y. (2016). Investigation and Research on the Evolution of Sichuan Pepper Varieties and Commercial Medicinal Materials. J. Chin. Med. Mater..

[3] Ding X., Liu N., Zhao Y., Su L., Sun J. (2020). Analysis of GC-MS Components of Volatile Oils from *Zanthoxylum bungeanum* from Different Producing Areas and Antibacterial Tests. J. Li Shizhen Tradit. Chin. Med..

[4] Wei L., Zong W., Zeng Q., Jiang Y., Zeng W., Chi H., Zhou Y., Chen M. (2021). Network Pharmacological Analysis and Experimental Verification of Anti-Inflammatory and Analgesic Effect of *Zanthoxyli pericarpium*. China J. Chin. Mater. Med..

[5] Zhang M., Wang D., Wei D., Ye X., He L., Pang W., Peng W., Wu C. (2022). Study on Quality Markers of *Zanthoxyli pericarpium* on Warming Middleenergizer to Alleviate Pain Based on Serum Medicinal Chemistry. Chin. Tradit. Herb. Drugs.

[6] Mewalal R., Rai D.K., Kainer D., Chen F., Külheim C., Peter G.F., Tuskan G.A. (2017). Plant-Derived Terpenes: A Feedstock for Specialty Biofuels. Trends Biotechnol..

[7] Couillaud J., Duquesne K., Iacazio G. (2022). Extension of the Terpene Chemical Space: The Very First Biosynthetic Steps. ChemBioChem.

[8] Shataer D., Chang Y., Obul M., Aierken K., Liu H. (2025). An Up-to-Date Review on the Classification, Pharmacology, and Production of Terpenes and Terpenoids. Curr. Org. Chem..

[9] Huang Z.-Y., Ye R.-Y., Yu H.-L., Li A.-T., Xu J.-H. (2021). Mining Methods and Typical Structural Mechanisms of Terpene Cyclases. Bioresour. Bioprocess..

[10] Huang Y., Xie F.J., Cao X., Li M.Y. (2021). Research Progress in Biosynthesis and Regulation of Plant Terpenoids. Biotechnol. Biotechnol. Equip..

[11] Devi M.L., Singh N.B., Sharma K.C., Rajashekar Y., Mishra A., Das S. (2023). Volatile Compound Profile Analysis of Seasonal Flower, Fruit, Leaf, and Stem of *Zanthoxylum armatum* DC. from Manipur Using HS-SPME-GC-MS. Chemosensors.

[12] Singh B., Sharma R.A. (2015). Plant Terpenes: Defense Responses, Phylogenetic Analysis, Regulation and Clinical Applications. 3 Biotech.

[13] Tang J., Liao Q., Zhang W., Tan S., Lan J., Li Z., Liu X. (2020). Comparative Study of Volatile Components in Fruits of Thorny and Non-Thorny Types of *Zanthoxylum schinifolium*. Food Sci. Technol. Res..

[14] Trung H.D., Thang T.D., Khôi N.K., Dai D.N., Ogunwande I.A. (2016). Chemical Constituents of Essential Oils from the Leaf, Flower and Fruit of *Zanthoxylum avicenna* (Lam.) DC (Rutaceae) from Vietnam. J. Essent. Oil Bear. Plants.

[15] Khamtache-Abderrahim S., Lequart-Pillon M., Gontier E., Gaillard I., Pilard S., Mathiron D., Djoudad-Kadji H., Maiza-Benabdesselam F. (2016). Isoquinoline Alkaloid Fractions of Fumaria Officinalis: Characterization and Evaluation of Their Antioxidant and Antibacterial Activities. Ind. Crops Prod..

[16] Di X., Ortega-Alarcon D., Kakumanu R., Iglesias-Fernandez J., Diaz L., Baidoo E.E.K., Velazquez-Campoy A., Rodríguez-Concepción M., Perez-Gil J. (2023). MEP Pathway Products Allosterically Promote Monomerization of Deoxy-D-Xylulose-5-Phosphate Synthase to Feedback-Regulate Their Supply. Plant Commun..

[17] Wei D., Zhao Y., Zhang M., Zhu L., Wang L., Yuan X., Wu C. (2021). The Volatile Oil of *Zanthoxylum bungeanum* Pericarp Improved the Hypothalamic-Pituitary-Adrenal Axis and Gut Microbiota to Attenuate Chronic Unpredictable Stress-Induced Anxiety Behavior in Rats. Drug Des. Devel. Ther..

[18] Jing N., Wang M., Gao M., Zhong Z., Ma Y., Wei A. (2021). Color Sensory Characteristics, Nutritional Components and Antioxidant Capacity of *Zanthoxylum bungeanum* Maxim. as Affected by Different Drying Methods. Ind. Crops Prod..

[19] Xu S., Yu L., Hou Y., Huang B., Wang H., Li D., Wang D. (2023). Chemical Composition, Chemotypic Characterization, and Histochemical Localization of Volatile Components in Different Cultivars of *Zanthoxylum bungeanum* Maxim. Leaves. J. Food Sci..

[20] Tang D., Liang Q., Zhang M., Li M., Zhang Q., Zhang S., Ai L., Wu C. (2022). Anti-Depression Effectiveness of Essential Oil from the Fruits of *Zanthoxylum bungeanum* Maxim. on Chronic Unpredictable Mild Stress-Induced Depression Behavior in Mice. Front. Pharmacol..

[21] Zhang W.-J., Guo S.-S., You C.-X., Geng Z.-F., Liang J.-Y., Deng Z.-W., Wang C.-F., Du S.-S., Wang Y.-Y. (2016). Chemical Composition of Essential Oils from *Zanthoxylum bungeanum* Maxim. and Their Bioactivities against *Lasioderma serricorne*. J. Oleo Sci..

[22] Zhang H., Guo Z., Wang X., Xian J., Zou L., Zheng C., Zhang J. (2022). Protective Mechanisms of *Zanthoxylum bungeanum* Essential Oil on DSS-Induced Ulcerative Colitis in Mice Based on a Colonic Mucosal Transcriptomic Approach. Food Funct..

[23] Zhou X.L., Chen L.L., Wang J.F. (2022). Study on the Antipruritic Mechanism of *Zanthoxylum bungeanum* and *Zanthoxylum schinifolium* Volatile Oil on Chronic Eczema Based on H1R and PAR-2 Mediated GRPR Pathway. Allergol. Immunopathol..

[24] Zheng T., Su K., Gao M., Zhang D., Chen X., Liu S. (2021). Chemotaxonomic Variation in Volatile Component Contents and Their Correlation between Climate Factors in Chinese Prickly Ash Peels (*Zanthoxylum bungeanum* Maxim.). Food Chem. X.

[25] Liang J., An Y., Hou Z., Wang X., Zhou F., Zhang J., Wang J. (2022). Acute Toxicity of *Zanthoxylum bungeanum* against Two Stored Product Insects and Synergistic Interactions between Two Major Compounds Limonene and Linalool. J. Environ. Sci. Health Part B.

[26] Tine Y., Diop A., Diatta W., Desjobert J.-M., Boye C.S.B., Costa J., Wélé A., Paolini J. (2017). Chemical Diversity and Antimicrobial Activity of Volatile Compounds from *Zanthoxylum zanthoxyloides*
LAM. According to Compound Classes, Plant Organs and Senegalese Sample Locations. Chem. Biodivers..

[27] Zhang M., Wei D., He L., Wang D., Wang L., Tang D., Zhao R., Ye X., Wu C., Peng W. (2022). Application of Response Surface Methodology (RSM) for Optimization of the Supercritical CO_2_ Extract of Oil from *Zanthoxylum bungeanum* Pericarp: Yield, Composition and Gastric Protective Effect. Food Chem. X.

[28] Zhu L., Wang L., Chen X., Peng W., Liu Y., Yu L., Liang F., Wu C. (2019). Comparative Studies on Flavor Substances of Leaves and Pericarps of *Zanthoxylum bungeanum* Maxim. at Different Harvest Periods. Trop. J. Pharm. Res..

[29] Karami-Osboo R., Miri R., Asadollahi M., Jassbi A.R. (2015). Comparison between Head-Space SPME and Hydrodistillation-GC-MS of the Volatiles of *Thymus daenensis*. J. Essent. Oil Bear. Plants.

[30] Liao S., Yang G., Huang S., Li B., Li A., Kan J. (2022). Chemical Composition of *Zanthoxylum schinifolium* Siebold & Zucc. Essential Oil and Evaluation of Its Antifungal Activity and Potential Modes of Action on Malassezia Restricta. Ind. Crops Prod..

[31] Zhang W., Tan S., Xi W., Yang J., Liao Q., Lan J., Lv Y., Tang J. (2019). Comparison of Volatile Components in Fresh and Dried *Zanthoxylum bungeanum* Maxim. Food Sci. Biotechnol..

[32] Liu S., Wang S., Song S., Zou Y., Wang J., Sun B. (2017). Characteristic Differences in Essential Oil Composition of Six *Zanthoxylum bungeanum* Maxim. (Rutaceae) Cultivars and Their Biological Significance. J. Zhejiang Univ.-Sci. B.

[33] Niu W., Tian H., Zhan P. (2022). The Effects of Pepper (*Zanthoxylum bungeanum*) from Different Production Areas on the Volatile Flavor Compounds of Fried Pepper Oils Based on HS-SPME–GC–MS and Multivariate Statistical Method. Molecules.

[34] Liu Y., Li Q., Yang W., Sun B., Zhou Y., Zheng Y., Huang M., Yang W. (2020). Characterization of the Potent Odorants in Zanthoxylum Armatum DC Prodr. Pericarp Oil by Application of Gas Chromatography–Mass Spectrometry–Olfactometry and Odor Activity Value. Food Chem..

[35] Liu J., Wang Y., Hou X., Cui Q., Wu H., Shen G., Luo Q., Li S., Liu X., Li M. (2023). Starch-Based Film Functionalized with *Zanthoxylum armatum* Essential Oil Improved the Shelf Life of Beef Sauce. LWT.

[36] Raguso R.A. (2016). More Lessons from Linalool: Insights Gained from a Ubiquitous Floral Volatile. Curr. Opin. Plant Biol..

[37] Nagegowda D.A., Gupta P. (2020). Advances in Biosynthesis, Regulation, and Metabolic Engineering of Plant Specialized Terpenoids. Plant Sci..

[38] Wang Z., He Z., Zhang D., Chen X., Li H. (2021). The Effect of Linalool, Limonene and Sabinene on the Thermal Stability and Structure of Rabbit Meat Myofibrillar Protein under Malondialdehyde-Induced Oxidative Stress. LWT.

[39] Wang L., Zhang J., Xu G., Guo Z., Wang J., Huang L., Wei L., Wang L., Zhang K., Li J. (2026). Linalool Disrupts *Escherichia coli* Biofilms via Dual Suppression of Motility and Adhesion. Front. Vet. Sci..

[40] Danna C., Malaspina P., Cornara L., Smeriglio A., Trombetta D., De Feo V., Vanin S. (2024). *Eucalyptus* Essential Oils in Pest Control: A Review of Chemical Composition and Applications against Insects and Mites. Crop Prot..

[41] Juergens L.J., Worth H., Juergens U.R. (2020). New Perspectives for Mucolytic, Anti-Inflammatory and Adjunctive Therapy with 1,8-Cineole in COPD and Asthma: Review on the New Therapeutic Approach. Adv. Ther..

[42] Zhang J., Pan S., Li Y., Diao X., Liu S. (2024). Nerolidol Inhibits Proliferation and Triggers ROS-Facilitated Apoptosis in Lung Carcinoma Cells via the Suppression of MAPK/STAT3/NF-κB and P13K/AKT Pathways. Adv. Clin. Exp. Med..

[43] Marhofer D., Jaksch W., Aigmüller T., Jochberger S., Urlesberger B., Pils K., Maier B., Likar R., Kayer B., Wallner R. (2021). Schmerztherapie in Der Schwangerschaft: Eine expertInnenbasierte Interdisziplinäre Konsensus-Empfehlung. Schmerz.

[44] Pourbagher Shahri A.M., Negah S.S., Moghbeli M., Saburi E., Forouzanfar F. (2025). A Review of the Anticancer Properties of Cedrol and Its Molecular Mechanisms. Anti-Cancer Agents Med. Chem..

[45] Nadanaka S., Tamura J., Kitagawa H. (2022). Chondroitin Sulfates Control Invasiveness of the Basal-like Breast Cancer Cell Line MDA-MB-231 through ROR1. Front. Oncol..

[46] Liang S., Hu W., Cheng W., Zhang S., Zou R. (2023). *Zanthoxylum bungeanum* Essential Oil: Extraction and Component Analysis for α-Glucosidase Inhibitory Activity and the Underlying Mechanism Based on Molecular Docking. Appl. Sci..

[47] Zeng L., Liu Y., Yuan Z., Wang Z. (2021). Formation and Physical Stability of *Zanthoxylum bungeanum* Essential Oil Based Nanoemulsions Co-Stabilized with Tea Saponin and Synthetic Surfactant. Molecules.

[48] Friščić M., Maleš Ž., Maleš I., Duka I., Radonić A., Mitić B., Hruševar D., Jurić S., Jerković I. (2024). Gas Chromatography–Mass Spectrometry Analysis of Volatile Organic Compounds from Three Endemic Iris Taxa: Headspace Solid-Phase Microextraction vs. Hydrodistillation. Molecules.

[49] Yuan R., Shi Y., Zhang J., Hu Q., Wei X., Luo C., Wu Y., Yang J., Yang M., Wang F. (2021). Study on the Chemical Constituents and Anti-Migraine Activity of Supercritical CO_2_ Extracts of *Zanthoxylum schinifolium*. Front. Pharmacol..

[50] Wu X., Yin J., Ding H., Li W., Han L., Yang W., Li F., Song X., Bie S., Gong X. (2022). The Discrimination and Characterization of Volatile Organic Compounds in Different Areas of *Zanthoxylum bungeanum* Pericarps and Leaves by HS-GC-IMS and HS-SPME-GC-MS. Foods.

[51] Kintamani E., Batubara I., Kusmana C., Tiryana T., Mirmanto E., Asoka S.F. (2023). Essential Oil Compounds of Andaliman (*Zanthoxylum acanthopodium* DC.) Fruit Varieties and Their Utilization as Skin Anti-Aging Using Molecular Docking. Life.

[52] Lei H., Wu J., Zhou R.J. (2017). Extraction and analysis of *Zanthoxylum bungeanum* essential oil by supercritical CO_2_ method optimized by response surface methodology and GC-MS. Curr. Top. Nutraceutical Res..

[53] Oh M., Chung M.S. (2014). Effects of Oils and Essential Oils from Seeds of *Zanthoxylum schinifolium* against Foodborne Viral Surrogates. Evid.-Based Complement. Alternat. Med..

[54] Kornpointner C., Sainz Martinez A., Marinovic S., Haselmair-Gosch C., Jamnik P., Schröder K., Löfke C., Halbwirth H. (2021). Chemical Composition and Antioxidant Potential of *Cannabis sativa* L. Roots. Ind. Crops Prod..

[55] Shah W.A., Dar M.Y., Rather M.A., Qurishi M.A. (2012). Comparison of Terpene Composition of *Skimmia laureola* Using Hydrodistillation and HS-SPME Techniques. J. Essent. Oil Bear. Plants.

[56] Yang X. (2008). Aroma Constituents and Alkylamides of Red and Green Huajiao (*Zanthoxylum bungeanum* and *Zanthoxylum schinifolium*). J. Agric. Food Chem..

[57] Satoh M., Kusumoto N., Matsui N., Makino R., Hashida K., Arai D., Iiduka Y., Ashitani T. (2022). Antitermitic and Antifungal Properties of Enantiopure Linalool and Furanoid Linalool Oxide Confirmed in *Lindera umbellata* Var. *Membranacea*. J. Wood Chem. Technol..

[58] Wu G., Wu H. (2014). Analgesia Synergism of Essential Oil from Pericarp of *Zanthoxylum schinifolium* and Verapamil. Evid.-Based Complement. Alternat. Med..

[59] Wang L., Wang Z., Li X., Zhang H., Zhou X., Zhang H. (2010). Analysis of Volatile Compounds in the Pericarp of *Zanthoxylum bungeanum* Maxim. by Ultrasonic Nebulization Extraction Coupled with Headspace Single-Drop Microextraction and GC–MS. Chromatographia.

[60] Bozova B., Gölükcü M., Giuffrè A.M. (2024). The Effect of Different Hydrodistillation Times on the Composition and Yield of Bergamot (*Citrus bergamia* Risso) Peel Essential Oil and a Comparison of the COLD-PRESSING Method. Flavour Fragr. J..

[61] Zhang Z., Shen P., Liu J., Gu C., Lu X., Li Y., Cao Y., Liu B., Fu Y., Zhang N. (2017). In Vivo Study of the Efficacy of the Essential Oil of *Zanthoxylum bungeanum* Pericarp in Dextran Sulfate Sodium-Induced Murine Experimental Colitis. J. Agric. Food Chem..

[62] Nunes J.D.A., Teixeira L.L., Nascimento W.M.O., Lopes D.C.F., Da Silva J.K.R., Pinto L.C., Santos P.V.L., Figueiredo P.L.B. (2025). Seasonal Variation on Chemical Composition and in Vitro Cytotoxic and Anti-Inflammatory Activities of *Myrciaria dubia* (Kunth) McVaugh Essential Oil from Amazon. Biochem. Syst. Ecol..

[63] Bisht A., Bahukhandi A., Trivedi V.L., Bhandari U., Attri D.C., Lohani H. (2026). Gender-Based Variation in the Essential Oil Composition of *Zanthoxylum armatum* DC. Across Different Populations in Uttarakhand. Natl. Acad. Sci. Lett..

[64] Gu W., Wei Y., Fu X., Gu R., Chen J., Jian J., Huang L., Yuan C., Guan W., Hao X. (2023). HS-SPME/GC×GC-TOFMS-Based Flavoromics and Antimicrobial Properties of the Aroma Components of *Zanthoxylum motuoense*. Foods.

[65] Ma Y., Tian J., Chen Y., Chen M., Liu Y., Wei A. (2021). Volatile Oil Profile of Prickly Ash (*Zanthoxylum*) Pericarps from Different Locations in China. Foods.

[66] Sriwichai T., Wisetkomolmat J., Pusadee T., Sringarm K., Duangmal K., Prasad S.K., Chuttong B., Sommano S.R. (2021). Aromatic Profile Variation of Essential Oil from Dried Makwhaen Fruit and Related Species. Plants.

[67] Xie G., Xie S., Du L., Chen C. (2026). Comparative Lipidomics Unveils Species-Specific Lipid Signatures in Three *Zanthoxylum* Species. Foods.

[68] Wu Y., Zhuo Z., Qian Q., Xu D. (2024). Chemotaxonomic Variation of Volatile Components in *Zanthoxylum bungeanum* Peel and Effects of Climate on Volatile Components. BMC Plant Biol..

[69] Xiao W., Guo J., Liao Y., Fu Y., Li Z., Zhang T., Liu T., Xiao Y., Shang X., Gao Z. (2026). The Role of Geography and Regional Climate in Shaping the Chemical Profiles and Biological Potentials of Citrus Essential Oils. Food Chem..

[70] Lan Y., Wu Q., Mao Y., Wang Q., An J., Chen Y., Wang W., Zhao B., Liu N., Zhang Y. (2014). Cytotoxicity and Enhancement Activity of Essential Oil from *Zanthoxylum bungeanum* Maxim. as a Natural Transdermal Penetration Enhancer. J. Zhejiang Univ. Sci. B.

[71] Huang X., Zhang W., Liao Y., Ye J., Xu F. (2024). Contemporary Understanding of Transcription Factor Regulation of Terpenoid Biosynthesis in Plants. Planta.

[72] Liu Y., Zhang Y., Wei X., Wu D., Dai J., Liu S., Qin W. (2021). Effect of Radio Frequency-Assisted Hot-Air Drying on Drying Kinetics and Quality of Sichuan Pepper (*Zanthoxylum bungeanum* Maxim.). LWT.

[73] Zhao C., Zhang F., Chen S., Hu W., Dong L., Zhao Y., Han M., Li Z. (2023). Effects of Drying Methods on the Quality of Hanyuan *Zanthoxylum bungeanum* Based on Physicochemical and Functional Metabolite Analysis. LWT.

[74] Suharta S., Hunaefi D., Wijaya C.H. (2021). Changes in Volatiles and Aroma Profile of Andaliman (*Zanthoxylum acanthopodium* DC.) upon Various Drying Techniques. Food Chem..

[75] Bhattacharjee S., Mohanty P., Sahu J.K., Sahu J.N. (2024). A Critical Review on Drying of Food Materials: Recent Progress and Key Challenges. Int. Commun. Heat Mass Transf..

[76] Lan Y., Li H., Chen Y., Zhang Y., Liu N., Zhang Q., Wu Q. (2014). Essential Oil from *Zanthoxylum bungeanum* Maxim. and Its Main Components Used as Transdermal Penetration Enhancers: A Comparative Study. J. Zhejiang Univ. Sci. B.

[77] Zhang H., Huang T., Liao X., Zhou Y., Chen S., Chen J., Xiong W. (2022). Extraction of Camphor Tree Essential Oil by Steam Distillation and Supercritical CO_2_ Extraction. Molecules.

[78] Hu F., Zhang A., Ji Z.-L., Thakur K., Zhang J.-G., Wei Z.-J. (2025). Effects of Different Extraction Methods on the Volatile Components and Numbing Substances in Red Huajiao (*Zanthoxylum bungeanum* Maxim.) and Green Huajiao (*Zanthoxylum armatum* DC.). Food Chem. X.

[79] Moreira R.C., De Melo R.P.F., Martínez J., Marostica Junior M.R., Pastore G.M., Zorn H., Bicas J.L. (2023). Supercritical CO_2_ as a Valuable Tool for Aroma Technology. J. Agric. Food Chem..

[80] Zeng Z., Mao Z., Liu Y., Chen M., Xu Z., Yan X., Xu G., Zhu W., Liu H., Ji Y. (2023). Functional Substances and Therapeutic Potential of Kumquat Essential Oil. Trends Food Sci. Technol..

[81] Qi J., Pan Z., Wang X., Zhang N., He G., Jiang X. (2024). Research Advances of *Zanthoxylum bungeanum* Maxim. Polyphenols in Inflammatory Diseases. Front. Immunol..

[82] Okagu I.U., Ndefo J.C., Aham E.C., Udenigwe C.C. (2021). *Zanthoxylum* Species: A Review of Traditional Uses, Phytochemistry and Pharmacology in Relation to Cancer, Infectious Diseases and Sickle Cell Anemia. Front. Pharmacol..

[83] Liao S., Yang G., Ou Y., Huang S., Li B., Li A., Kan J. (2022). Inhibitory Impacts of Essential Oil (*Zanthoxylum schinifolium* Sieb. et Zucc) on the Growth of Staphylococcus Epidermidis. Food Biosci..

[84] Shu Z., Zhang X., Song S., Fan J. (2025). Antibacterial Activity and Mechanism of Ludian Green Pepper (*Z. schinifolium*) Essential Oils against *Staphylococcus aureus*. Sci. Technol. Food Ind..

[85] Wu K., Zhao X., Duan X., Chai X., Yu H., Liu X., Fan Y. (2020). Antibacterial Activity and Mechanism of Action of Vapor-Phase Linalool. Food Sci..

[86] Krapež P., Lunder M., Oder M., Fink R. (2024). Evaluation of the In Vitro Disinfection Potential of the Phytochemicals Linalool and Citronellal against Biofilms Formed by *Escherichia coli* and *Staphylococcus aureus*. Processes.

[87] Wang Q.Y., Jing X.H. (2018). Research Progress of the Chemical Compositions, Extraction Methodsand Antibacterial Activities of Essential Oil from *Zanthoxylum bungeanum*. China Condiment.

[88] Li X.-D., Xue H.-L. (2014). Antifungal Activity of the Essential Oil of *Zanthoxylum bungeanum* and Its Major Constituent on Fusarium Sulphureum and Dry Rot of Potato Tubers. Phytoparasitica.

[89] Jiang Y.H., Zhao Y.T., Xin W.G., Liang M., Song J.J., Suo H.Y. (2025). Synergistic Inactivation Effect and Mechanism of Ultrasound Combined with *Zanthoxylum schinifolium* Essential Oil Nanoemulsions against *Escherichia coli* O157:H7 and Its Application on Fresh-Cut Cucumber. Int. J. Food Microbiol..

[90] Khruengsai S., Sripahco T., Pripdeevech P. (2023). Antibacterial Activity and Synergic Effects of the Essential Oils of *Amomum verum* Blackw and *Zanthoxylum limonella* (Dennst.) Alston. Arch. Microbiol..

[91] Li Q., Chen Z., Zeng L., Bi Y., Kong F., Wang Z., Tan S. (2023). Characterization, in-Vitro Digestion, Antioxidant, Anti-Hyperlipidemic and Antibacterial Activities of *Zanthoxylum bungeanum* Maxim Essential Oil Nano-Emulsion. Food Biosci..

[92] Ali S.S., Al-Tohamy R., Al-Zahrani M., Badr A., Sun J. (2025). Essential Oils and Plant-Derived Bioactive Compounds: A Comprehensive Review of Their Therapeutic Potential, Mechanisms of Action, and Advances in Extraction Technologies. Phytochem. Rev..

[93] Wang Z., He Z., Zhang D., Chen X., Li H. (2022). Effect of Pepper (*Zanthoxylum bungeanum* Maxim.) Essential Oil on Quality Changes in Rabbit Meat Patty during Chilled Storage. J. Food Sci. Technol..

[94] Zhang H., Lang X., Zhang Y., Wang C. (2022). Distribution of Bacteria in Different Regions of the Small Intestine with *Zanthoxylum bungeanum* Essential Oil Supplement in Small-Tailed Han Sheep. Front. Microbiol..

[95] Xia X. (2018). Efficacy Observation of External Application of Sichuan Pepper Oil in the Treatment of Chronic Localized Eczema. Master’s Thesis.

[96] Zhong H., Han L., Lu R.Y., Wang Y. (2022). Antifungal and Immunomodulatory Ingredients from Traditional Chinese Medicine. Antibiotics.

[97] Liu J., Zheng F., Li Y., Wang W., Qi C., Yao Q. (2018). Progress on Anti-Infectious Activities of Plants from Genus Zanthoxylum. Chin. Tradit. Herb. Drugs.

[98] Imaz L., Aliakbarlu J., Lin L. (2023). Combined Antifungal Effects of the Vapor Phases of *Zataria multiflora* and *Cinnamomum zeylanicum* Essential Oils against *Aspergillus flavus* and *Penicillium citrinum* in Vitro and Cheese. Food Sci. Nutr..

[99] Tahmasebi M., Golmohammadi A., Nematollahzadeh A., Davari M., Chamani E. (2020). Control of Nectarine Fruits Postharvest Fungal Rots Caused by *Botrytis Cinerea* and *Rhizopus Stolonifer* via Some Essential Oils. J. Food Sci. Technol..

[100] Yuan J.L. (2010). The Anti-Inflammatory and Analgesic Effects of Zanthoxylum Volatile Oil. J. Chin. Med. Mater..

[101] Zhang M., Wang J., Zhu L., Li T., Jiang W., Zhou J., Peng W., Wu C. (2017). *Zanthoxylum bungeanum* Maxim. (Rutaceae): A Systematic Review of Its Traditional Uses, Botany, Phytochemistry, Pharmacology, Pharmacokinetics, and Toxicology. Int. J. Mol. Sci..

[102] Li X.-Q., Kang R., Huo J.-C., Xie Y.-H., Wang S.-W., Cao W. (2017). Wound-healing activity of *Zanthoxylum bungeanum* maxim seed oil on experimentally burned rats. Pharmacogn. Mag..

[103] Xu T., Lin M., He Y., Gu Z. (2020). Regulatory Effect of *Zanthoxylum bungeanum* Seed Oil on Wound Healing and Serum Inflammatory Factors in Rats with Burn Injury. Chin. J. Clin. Pharmacol..

[104] Hu Q., Peng Y., Li M., Wen X., Ni Y. (2024). Research Progress on Bioactivities and Mechanisms of *Zanthoxylum bungeanum* Oils. J. Chin. Cereals Oils Assoc..

[105] Luo F.H., Chen Z.H., Zeng F.F., Yang X., Li J.J., Zhang F.X., Shi W. (2025). Botany, Phytochemistry, Pharmacologic Activities, Traditional Applications, Pharmacokinetics, Quality Control and Toxicity of *Zanthoxyli* Radix: An Updated Review. J. Ethnopharmacol..

[106] Yuan T.N., Wang Y.L., Wang Y.Z. (2008). Primary Study of the Anti-Tumor Effect and Its Mechanism of *Zanthoxylum* in vivo and in vitro. J. Li-Shizhen Tradit. Chin. Med..

[107] Wu Y., Li Z., Bai J., Luo T., Xu X., Lu L., Han X., Wong J. (2023). Analysis of Chemical Constituents and Activity of Essential Oil from Sichuan Pepper from Different Origins. China Food Addit..

[108] Yuan T.N., Wang Y.L., Wang Y.Z. (2008). Study on the Effect of Zanthoxylum Volatile Oil against Cervical Cancer HeLa Cells. J. Hubei Minzu Univ. Ed..

[109] Han S.N., Li Y., Zhang X., Jiang J.L. (2014). Extraction and Antitumor Activity of Essential Oil from *Zanthoxylum bungeanum* Seeds. Food Sci..

[110] Pang W., Liu S., He F., Li X., Saira B., Zheng T., Chen J., Dong K., Pei X. (2019). Anticancer Activities of *Zanthoxylum bungeanum* Seed Oil on Malignant Melanoma. J. Ethnopharmacol..

[111] Bai Y., Hou J., Zhang X.T., Gao J.P., Zhou J.T. (2021). *Zanthoxylum bungeanum* Seed Oil Elicits Autophagy and Apoptosis in Human Laryngeal Tumor Cells via PI3K/AKT/mTOR Signaling Pathway. Anti-Cancer Agents Med. Chem..

[112] Almalki S.G. (2023). The Pathophysiology of the Cell Cycle in Cancer and Treatment Strategies Using Various Cell Cycle Checkpoint Inhibitors. Pathol. Res. Pract..

[113] Wen T.R., Wu H., Zhu J., Zheng C., You F.M. (2022). Effect of Atomization Inhalation of Huajiao (*Zanthoxylum bungeanum*) Essential Oil on Inflammatory and Cancer Transformation in CAC Mice and Its Mechanism. Chin. Arch. Tradit. Chin. Med..

[114] Azadi M., Jamali T., Kianmehr Z., Kavoosi G., Ardestani S.K. (2020). In-vitro (2D and 3D Cultures) and in-vivo Cytotoxic Properties of *Zataria multiflora* Essential Oil (ZEO) Emulsion in Breast and Cervical Cancer Cells along with the Investigation of Immunomodulatory Potential. J. Ethnopharmacol..

[115] Gu Z.Y., Liu D.W., Xing J.G., Yang W.J. (2011). Quality Standard of Anaerfujie Vagina Effervescent Tablets. Chin. J. Exp. Tradit. Med. Formulae.

[116] Cheng Z.M., Chen Y.R., Wang J.H., Liu D.M., Fang F., Zhang B., Mu M.W., Xie Y.F., Yi J.Z. (2022). Antibacterial Activity of Essential Oils from *Zanthoxylum schinifolium* Siebold & Zucc. against Cariogenic Bacteria. Food Sci..

[117] Bedini S., Djebbi T., Ascrizzi R., Farina P., Pieracci Y., Echeverría M.C., Flamini G., Trusendi F., Ortega S., Chiliquinga A. (2024). Repellence and Attractiveness: The Hormetic Effect of Aromatic Plant Essential Oils on Insect Behavior. Ind. Crops Prod..

[118] Feng R., Ma Z., Bai J., Fan J., He C., Wang J., Zhang J., Liang J., Zhou F. (2025). Bioactivity of Essential Oils Extracted from Four Varieties of *Zanthoxylum bungeanum* against Insects and Dominant Spoilage Fungi in *Codonopsis pilosulae*. S. Afr. J. Bot..

[119] Okagu I.U., Okeke E.S., Ezeorba W.C.F., Ndefo J.C., Ezeorba T.P.C. (2023). Overhauling the Ecotoxicological Impact of Synthetic Pesticides Using Plants’ Natural Products: A Focus on *Zanthoxylum metabolites*. Environ. Sci. Pollut. Res..

[120] Zhao X., Wang J. (2013). Study on the Incubation Inhibition of Pepper Essential Oils on Tribolium Eggs. J. Chin. Cereals Oils Assoc..

[121] Kim K.-H., Yi C.-G., Ahn Y.-J., Kim S.I., Lee S.-G., Kim J.-R. (2015). Fumigant Toxicity of Basil Oil Compounds and Related Compounds to *Thrips palmi* and *Orius strigicollis*. Pest Manag. Sci..

[122] Luo Z., Liu X., Xu C., Yan Y., Fu M., Peng M., Deng T., Yang J., Qin R. (2026). Exploring the Mechanism of *Zanthoxylum bungeanum* Essential Oil in Alleviating Acute Pruritus in Rats Based on Network Pharmacology, Molecular Docking, and Experimental Pharmacology. J. Ethnopharmacol..

[123] Yan C., Wang Y., He F.T., Zheng T.L., Li Y., Pei X.F. (2019). Inhibitory Effect of *Zanthoxylum bungeanum* Essential Oil on Cell Inflammation Caused by P. Acnes. Mod. Prev. Med..

[124] Wang Y., Chen Y.H., Pang W.W., Chen J.Y., Zheng T.L., Zhou T., Pei X.F. (2017). Antimicrobial Effect and the Related Mechanism of the Extracts from *Zanthoxylum bungeanum* against Acne-Inducing Bacteria. Mod. Prev. Med..

[125] Shi Y.M. (2023). Preparation, Characterization and Application Research of *Zanthoxylum* Oil Nanoemulsion. Master’s Thesis.

[126] Souto E.B., Macedo A.S., Dias-Ferreira J., Cano A., Zielińska A., Matos C.M. (2021). Elastic and Ultradeformable Liposomes for Transdermal Delivery of Active Pharmaceutical Ingredients (APIs). Int. J. Mol. Sci..

[127] Tu H. (2025). Preparation and Performance Study of *Zanthoxylum armatum* Essential Oil Inclusion Compounds Based on Target Enzyme Inhibition. Master’s Thesis.

[128] Zhang J.M. (2024). Preparation and Performance Study of *Zanthoxylum* Oil Essential Oil Microcapsules. Master’s Thesis.

[129] Ez-Zoubi A., Zaroual H., Zoubi Y.E., Fadil M., Farah A. (2024). Inclusion Complex Essential Oil into Cyclodextrins and Its Optimization via Experimental Designs: A Review. Chem. Pap..

[130] Liu Z., Liu H., Cheng J., Wang H., Yang Y., Ye J., Liu Y. (2024). Strategies and Opportunities of Micro/Nano Delivery Systems for Targeted Therapy of Ulcerative Colitis: Focus on Underlying Mechanisms and Future Perspectives. Chin. Chem. Lett..

[131] Dashipour A., Razavilar V., Hosseini H., Shojaee-Aliabadi S., German J.B., Ghanati K., Khakpour M., Khaksar R. (2015). Antioxidant and Antimicrobial Carboxymethyl Cellulose Films Containing *Zataria multiflora* Essential Oil. Int. J. Biol. Macromol..

[132] Sharma S., Mulrey L., Byrne M., Jaiswal A.K., Jaiswal S. (2022). Encapsulation of Essential Oils in Nanocarriers for Active Food Packaging. Foods.

[133] Potluri A., Sk A.S., Rallapally N., Durrivel S., Harish G. (2013). A Review on Herbs Used in Anti-Dandruff Shampoo and Its Evaluation Parameters. Indo Am. J. Pharm. Res..

[134] Kashiri M., Cerisuelo J.P., Domínguez I., López-Carballo G., Muriel-Gallet V., Gavara R., Hernández-Muñoz P. (2017). Zein Films and Coatings as Carriers and Release Systems of *Zataria multiflora* Boiss. Essential Oil for Antimicrobial Food Packaging. Food Hydrocoll..

[135] Zhao J., Chen J., Liu T., Zhang D., Su C., Chi H., Chen H., Tang J., Zhang X. (2026). Encapsulation of *Zanthoxylum schinifolium* Essential Oil by Complex Coacervation with Whey Protein Isolate and Gum Arabic for Improved Stability and Controlled Release. Colloids Surf. Physicochem. Eng. Asp..

[136] Liu B., Sun J., Han L., Yin Y., Meng X. (2025). Preparation of Dihydroquercetin Microcapsules with Sodium Starch Octenyl Succinate and Maltodextrin as Wall Materials to Improve Stability, Solubility and Bioaccessibility. Food Chem..

[137] Chen Z., Tian W., Qin X., Wang H., Tan L., Liu X. (2024). Chitosan/Oxidized Konjac Glucomannan Films Incorporated with *Zanthoxylum bungeanum* Essential Oil: A Novel Approach for Extending the Shelf Life of Meat. Int. J. Biol. Macromol..

[138] Moradinezhad F., Aliabadi M., Ansarifar E. (2024). Zein Multilayer Electrospun Nanofibers Contain Essential Oil: Release Kinetic, Functional Effectiveness, and Application to Fruit Preservation. Foods.

[139] Kalateh-Seifari F., Ahari H., Moradi S. (2025). A Review on the Food-Based Applications of Nanometric Plant-Based Essential Oils: Nanoencapsulation and Nanoemulsion Production Challenges. Food Bioprocess Technol..

[140] Yang T., Júnior L.M., Vieira R.P., Ananthi P., Li S., Raj KV A., Roy S., Xia G. (2026). Hydrocolloid-Based Stimuli-Responsive Nanofiber Films: Precision-Controlled Release of Essential Oils for Sustainable Food Packaging. Curr. Res. Food Sci..

[141] Li H., Zhao L., Dai Q., Mo H., Liu Z., Pu H., Zhu X., Yao L., Xu D., Hu L. (2023). Blended Cumin/Zanthoxylum Essential Oil Improve the Antibacterial, Fresh-Keeping Performance and Flavor of Chilled Fresh Mutton. Meat Sci..

[142] Khayyat S.A., Roselin L.S. (2018). Recent Progress in Photochemical Reaction on Main Components of Some Essential Oils. J. Saudi Chem. Soc..

[143] Yang X., Liang Y., Li K., Hu Q., He J., Xie J. (2025). Advances in Microencapsulation of Flavor Substances: Preparation Techniques, Wall Material Selection, Characterization Methods, and Applications. J. Agric. Food Chem..

[144] Gong X., Ma X.J., Kang F.E., Jin Q. (2023). Research Progress on Extraction and Application of Bioactive Components in *Zanthoxylum* Essential Oil. J. Cold-Arid Agric. Sci..

